# The Heat Shock Transcription Factor HsfA Plays a Role in Membrane Lipids Biosynthesis Connecting Thermotolerance and Unsaturated Fatty Acid Metabolism in Aspergillus fumigatus

**DOI:** 10.1128/spectrum.01627-23

**Published:** 2023-05-17

**Authors:** João Henrique Tadini Marilhano Fabri, Marina Campos Rocha, Caroline Mota Fernandes, Jonatas Erick Maimoni Campanella, Anderson Ferreira da Cunha, Maurizio Del Poeta, Iran Malavazi

**Affiliations:** a Departamento de Genética e Evolução, Centro de Ciências Biológicas e da Saúde, Universidade Federal de São Carlos, São Carlos, São Paulo, Brazil; b Department of Microbiology and Immunology, Stony Brook University, Stony Brook, New York, USA; c Division of Infectious Diseases, School of Medicine, Stony Brook University, Stony Brook, New York, USA; d Institute of Chemical Biology and Drug Discovery, Stony Brook University, Stony Brook, New York, USA; e Veterans Administration Medical Center, Northport, New York, USA; Universidade de Sao Paulo

**Keywords:** *Aspergillus fumigatus*, *hsfA*, *sdeA*, *hsp90*, unsaturated fatty acid, phospholipid, sphingolipid, heat shock

## Abstract

Thermotolerance is a remarkable virulence attribute of Aspergillus fumigatus, but the consequences of heat shock (HS) to the cell membrane of this fungus are unknown, although this structure is one of the first to detect changes in ambient temperature that imposes on the cell a prompt adaptative response. Under high-temperature stress, fungi trigger the HS response controlled by heat shock transcription factors, such as HsfA, which regulates the expression of heat shock proteins. In yeast, smaller amounts of phospholipids with unsaturated fatty acid (FA) chains are synthesized in response to HS, directly affecting plasma membrane composition. The addition of double bonds in saturated FA is catalyzed by Δ9-fatty acid desaturases, whose expression is temperature-modulated. However, the relationship between HS and saturated/unsaturated FA balance in membrane lipids of A. fumigatus in response to HS has not been investigated. Here, we found that HsfA responds to plasma membrane stress and has a role in sphingolipid and phospholipid unsaturated biosynthesis. In addition, we studied the A. fumigatus Δ9-fatty acid desaturase *sdeA* and discovered that this gene is essential and required for unsaturated FA biosynthesis, although it did not directly affect the total levels of phospholipids and sphingolipids. *sdeA* depletion significantly sensitizes mature A. fumigatus biofilms to caspofungin. Also, we demonstrate that *hsfA* controls *sdeA* expression, while SdeA and Hsp90 physically interact. Our results suggest that HsfA is required for the adaptation of the fungal plasma membrane to HS and point out a sharp relationship between thermotolerance and FA metabolism in A. fumigatus.

**IMPORTANCE** Aspergillus fumigatus causes invasive pulmonary aspergillosis, a life-threatening infection accounting for high mortality rates in immunocompromised patients. The ability of this organism to grow at elevated temperatures is long recognized as an essential attribute for this mold to cause disease. A. fumigatus responds to heat stress by activating heat shock transcription factors and chaperones to orchestrate cellular responses that protect the fungus against damage caused by heat. Concomitantly, the cell membrane must adapt to heat and maintain physical and chemical properties such as the balance between saturated/unsaturated fatty acids. However, how A. fumigatus connects these two physiological responses is unclear. Here, we explain that HsfA affects the synthesis of complex membrane lipids such as phospholipids and sphingolipids and controls the enzyme SdeA, which produces monounsaturated fatty acids, raw material for membrane lipids. These findings suggest that forced dysregulation of saturated/unsaturated fatty acid balance might represent novel strategies for antifungal therapy.

## INTRODUCTION

Despite the increasing number of global cases, fungal illnesses are usually underdiagnosed and neglected while associated with high economic burden (1, 2). The saprophytic mold Aspergillus fumigatus is one the most important human fungal pathogens and the main one responsible for causing a broad spectrum of clinical diseases, including invasive pulmonary aspergillosis (IPA), a deadly infection associated with high mortality rates in immunosuppressed patients (3, 4).

Although several molecular mechanisms that control A. fumigatus germination, hyphal morphology, biofilm formation, and their association to virulence have been elucidated, the heat-tolerant functional changes and the plasma membrane composition have not been fully addressed so far in this fungus. As a dynamic and fluid bilayer, the plasma membrane is a vital structure that allows various cellular functions in the fungal cells, including secretion and transport, signal transduction, apical growth, and, importantly, cell wall biosynthesis, since many cell wall biosynthetic enzymes are attached to the cell membrane (5, 6). Apart from the protein constituents, phospholipids, sphingolipids, and sterols are the main components of the fungal plasma membrane (7). These molecules are considered regulators of pathogenesis as they have been associated with drug resistance, biofilm formation, and release of extracellular vesicles with immunomodulatory properties in pathogenic fungi (8, 9). Not surprisingly, the plasma membrane is an important target for antifungal chemotherapy. The polyene amphotericin B (AMB) and the triazole voriconazole have been used as the first line of therapy against IPA (10). Nevertheless, due to the structural similarity between the AMB target ergosterol and the mammalian membrane sterol counterpart, AMB is notorious for causing toxicity to the host (11). In addition, long-term azole therapies lead to the emergence of resistant strains (12, 13).

Thermotolerance is a phenotypic trait long reported as an important virulence determinant in A. fumigatus (14, 15). As a thermophilic species, this fungus displays a robust heat shock (HS) response that potentially endures conidia germination and subsequent biofilm formation in the lungs under a sustained HS condition during infection. In fungi and other eukaryotes, the HS response initiates with the activation of heat shock transcription factors (HSFs), which induce the expression of heat shock proteins (HSPs) and other genes related to thermal adaptation (16–19). During this process, the plasma membrane is one of the first structures to detect ambient temperature fluctuations, changing its fluidity and other physical and chemical properties (20–22). However, whether this adaptation requires the activation of HSFs and how it affects the lipid composition of the cell membrane remains not fully elucidated. The balance between saturated and unsaturated fatty acids (FAs) ultimately dictates the fluid state of the biological membrane during temperature variations (20, 21, 23–25). Among other constituents, phospholipid and sphingolipid composition dictates the plasma membrane fluidity because the saturation and the size of the FAs forming such lipids increase at higher temperatures, resulting in less fluid cell membrane in fungi (20, 24, 26, 27).

In yeast, monounsaturated FA synthesis is catalyzed by an enzyme belonging to the class of Δ9-fatty acid desaturases, encoded by the essential gene *OLE1* (23, 28, 29). This enzyme has palmitoyl (16:0) and stearoyl (18:0) coenzyme A (CoA) as natural substrates and generates palmitoleic (16:1) and oleic (18:1) acids (29). The *OLE1* homolog in the fungal pathogen Candida albicans is also an essential gene required for aerobic hyphal growth, whereas the *sdeA*^OLE1^ deletion strain in the model organism Aspergillus nidulans is viable (30, 31). Previous studies have shown that *OLE1* is required for HS response and adaptation. Saccharomyces cerevisiae *OLE1* transcription increases in response to low temperature (32, 33), and interestingly, C. albicans *OLE1* depletion prevents complete activation of Hsf1 in response to heat stress (24). Moreover, *hsp82*, a chaperone from the Hsp90 class and *hsp70* gene expression in Histoplasma capsulatum are also modulated by the level of *OLE1* expression and by the supplementation of saturated or unsaturated FA in the culture medium (34, 35). However, the effects of genetic depletion of the *HSF1* and *OLE1* homologs in A. fumigatus and the contribution of these genes to the changes in cell membrane fluidity and chemical diversity of saturated and unsaturated FAs are unknown.

In this study, we found that HsfA has a role in the regulation of phospholipid and sphingolipid biosynthesis, and the depletion of *hsfA* causes an accumulation of phospholipids and sphingolipids during the HS. We show that the *sdeA*^OLE1^ homolog is an essential gene in A. fumigatus, and phenotypic analyses confirmed that *sdeA* is required for monounsaturated FA biosynthesis. Finally, we show that *hsfA* modulates SdeA protein expression, while SdeA modulates Hsp90 expression and physically interacts with this chaperone. Our results suggest that thermotolerance and plasma membrane metabolism are linked in A. fumigatus through HsfA and SdeA functions.

## RESULTS

### HsfA plays a role in lipid biosynthesis.

Recently, we demonstrated that the HS transcription factor HsfA is crucial for adaptation to HS and cell wall integrity (CWI) in A. fumigatus (36). In addition, we showed that *hsfA* depletion led to altered expression of genes involved in lipid biosynthesis (36). To probe further into the contribution of *hsfA* in lipids biosynthesis, we ran an additional RNA-sequencing (RNA-seq) analysis comparing the wild-type strain and the *xylP*::*hsfA* conditional mutant. In this mutant, *hsfA* expression is under the control of the xylose reductase gene promoter (*xylP*) from Penicillium chrysogenum, which can be repressed by glucose or induced by xylose (36, 37). For the RNA-seq analysis, we cultured the strains in the presence of 1% xylose, which causes overexpression of *hsfA* (~4- to 5-fold) compared to the wild-type strain, as previously reported (36). The rationale behind this experiment was to evidence genes whose mRNA abundance changes when HsfA is maintained at high levels in the cell at standard nonstressing conditions (30°C). Gene Ontology (GO) analysis of the differentially regulated genes in the *xylP*::*hsfA* strain showed that *hsfA* overexpression caused the enrichment of genes belonging to the biological processes such as response to heat (e.g., *hsp60*, *hsp90*), CWI maintenance (e.g., *agsB*, *agsC*, *gel1*, *gel3*), and trehalose biosynthesis (e.g., *tpsB*, *tpsD*), suggesting that induction of *hsfA* expression without perceptible HS is sufficient to trigger a HS-like response (File S1 [https://doi.org/10.6084/m9.figshare.22354441]). It is noteworthy that the GO analysis also pointed out the repression of genes belonging to the FA biosynthetic process, especially those responsible for the unsaturated FA synthesis (Table 1), such as the putative Δ9-fatty acid desaturases (Afu7g05920 and Afu7g05350) and the oleate Δ12-desaturase (Afu6g03050).

**TABLE 1:** Selected modulated genes enriched in GO categories related to lipid metabolism^*a*^

GO	*P* value	Log^2^ fold	Gene ID (A1163 strain)	Gene ID (Af293 strain)	Annotation (FungiDB)
Unsaturated fatty acid biosynthetic process (GO:0006636)^*b*^	0.0124	−1.1	AFUB_091500	Afu7g05920	Ortholog(s) have stearoyl-CoA 9-desaturase activity (*sdeA*)
		−1.2	AFUB_017380	Afu2g00320	Putative sterol Δ5,6-desaturase (*erg3*)
		−3.5	AFUB_095250	Afu6g03050	Oleate Δ12 desataurase
		−1.6	AFUB_090930^*c*^	Afu7g05350	Putative Δ9 fatty acid desaturase with a predicted role in fatty acid biosynthesis
Fatty acid biosynthetic process (GO:0006633)^*b*^	0.0172	−1.1	AFUB_016240	Afu1g16850	Putative sphinganine hydroxylase with a predicted role in the hydroxylation of DHS at position C-4 (*sur2*)
		−1.4	AFUB_037060	Afu3g12120	Putative fatty acid oxygenase (*ppoC*)
		−1.4	AFUB_096730	Afu6g00740	Has domain(s) with predicted iron ion binding, oxidoreductase activity, and role in fatty acid biosynthetic process, oxidation-reduction process
		−1.7	AFUB_084150	Afu8g02440	C-4 methyl sterol oxidase, putative (*erg25*)
		−2.8	AFUB_067850	Afu4g10770	Psi-producing oxygenase A (*ppoA*)
		−2.2	AFUB_044650	Afu5g09970	Has domain(s) with predicted FAD binding, oleate hydratase activity, and role in fatty acid metabolic process
		3.00	AFUB_100690	Afu4g00180	Fatty acid 8,11-diol synthase (*ppoB*)

a Selected modulated genes (log_2_FC ≥ 1.0 or log_2_FC ≤ –1.0) were enriched in GO categories related to lipid metabolism. The *xylP*::*hsfA* strain at 30°C in the presence of xylose 1% (*hsfA* overexpression) is compared to the wild-type strain under the same conditions. DHS, dihydrosphingosine; FAD, Flavin Adenine Dinucleotide; GO, Gene Ontology.

b Biological Process (BP). For the full BP GO terms list, refer to File S1 (https://doi.org/10.6084/m9.figshare.22354441).

c Annotated as a pseudogene in A1163 strain. Has domain(s) with predicted heme-binding activity. Not described as fatty acid desaturase by Tang et al. (56).

**FIG 1:**
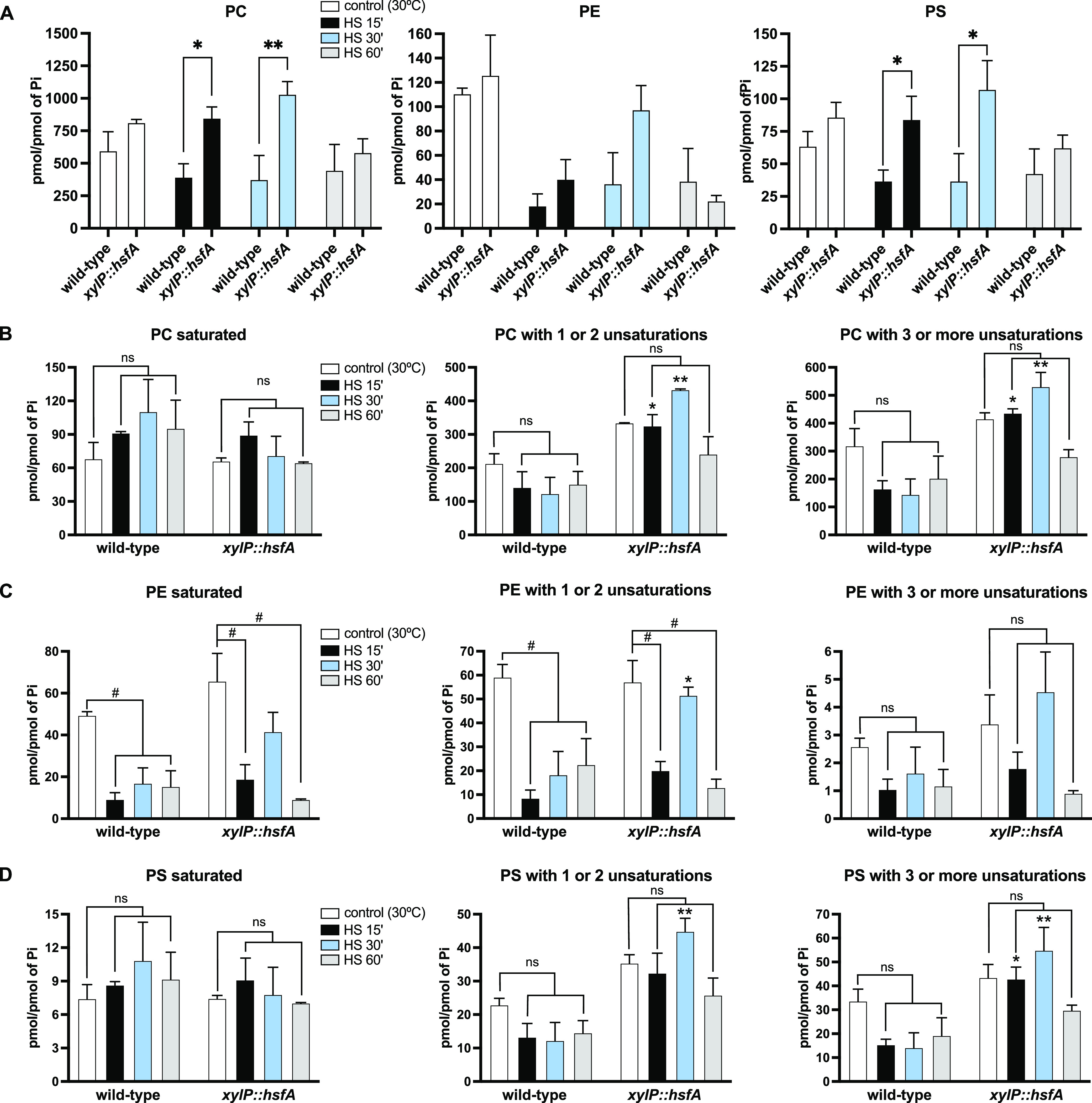
The *hsfA* loss of function alters the proportion of unsaturated phospholipids during heat shock (HS). (A) General proportion of the phospholipids classes: phosphatidylcholine (PC), phosphatidylethanolamine (PE), and phosphatidylserine (PS) measured in the wild-type and *xylP*::*hsfA* strains. The strains were grown in liquid minimal medium (MM) (1% glucose) supplemented with xylose 1% for 24 h at 30°C to allow growth. Subsequently, the mycelia were washed twice with MM and incubated for 4 h at 30°C in MM (glucose 1%; no xylose) for *xylP* repression. HS was induced by transferring the mycelia to fresh preheated MM (glucose 1%; no xylose) for an additional 15, 30, and 60 min of incubation at 48°C. The control was left at 30°C. (B to D) Levels of the three phospholipids classes grouped according to the degree of saturation of the fatty acid chains: saturated lipids, lipids with one or two unsaturations, and lipids with three or more unsaturations. Lipids were measured by mass spectrometry, and the values were normalized by the inorganic phosphate (P_i_) levels. *, *P ≤ *0.05; **, *P ≤ *0.01 (two-way analysis of variance [ANOVA] with Sidak’s post-test compared to wild-type strain in the same condition). Bars superscripted with # (*P ≤ *0.05) indicate significant comparisons within the same strain to evidence the HS effect. ns, nonsignificant.

Given these results, we decided to investigate the role of HsfA in plasma membrane homeostasis by subjecting the *xylP*::*hsfA* strain to a mass spectrometry analysis aiming to disclose the global responses in terms of phospholipids accumulation caused by the perturbation of the regulatory network of this transcription factor. To this end, the wild-type and *xylP*::*hsfA* strains were grown in liquid minimal medium (MM) (1% glucose) supplemented with xylose 1% for 24 h at 30°C to allow vegetative growth. Subsequently, the mycelia were washed twice with prewarmed MM and incubated for 4 h at 30°C in MM (glucose 1%; no xylose) for *xylP*::*hsfA* repression. Next, HS was induced by transferring the mycelia to fresh preheated MM (glucose 1%; no xylose) at 48°C for an additional 15, 30, and 60 min of incubation at 48°C. The control was left at 30°C. The levels of the top three most abundant phospholipids in fungal species, i.e., phosphatidylcholine (PC), phosphatidylethanolamine (PE), and phosphatidylserine (PS) (38, 39) in the wild-type and *xylP*::*hsfA* strains are shown in Fig. 1A. Similar to other fungal species, PC is also the most abundant phospholipid in A. fumigatus. We observed that the overall amount of the three types of phospholipids decreased in the wild-type strain during the HS, consistent with previous reports from other fungal species subjected to HS (40–43). For instance, PC significantly decreased ~33.7% in the wild-type strain after 15 min of HS (599.9 to 397.59 pmol/P_i_), while PE and PS concentrations dropped 83.2% (111.00 to 18.68 pmol/P_i_) and 41.7% (63.91 to 37.24 pmol/P_i_), respectively (Fig. 1A). Conversely, *xylP*::*hsfA* mutant sustains the basal levels of phospholipids observed for the control condition leading to significant differences between the two strains in pairwise comparisons, which are more evident for PC and PS after 15 and 30 min of HS (asterisks in Fig. 1A). This result is likely driven by the above-mentioned significant reduction in the phospholipid concentration in the wild-type strain. Despite that, these results suggest a possible inhibitory role of HsfA on phospholipid biosynthesis, which is more evident during the HS.

When the temperature rises, one of the first responses associated with the cell membrane is the decrease in unsaturated FA synthesis to maintain lower fluidity and sustain plasma membrane properties (44). To gather information about the chemical diversity of the phospholipids that accumulated in our experimental conditions, the quantification results were categorized based on the saturation level of the FA comprising each phospholipid (Fig. 1B to D). The accumulation pattern of saturated phospholipids in both strains was similar pre- and post-HS. However, while saturated PC and PS had a nonsignificant trend toward increasing, saturated PE significantly decreased throughout the HS in both strains (#, Fig. 1C). When the levels of unsaturated phospholipids were compared between wild-type and *xylP*::*hsfA*, there was a significant increase in PC and PS concentration, especially after 15 and 30 min post-HS (asterisks, Fig. 1B and D). The heat maps detail the PC, PE, and PS species regulated by HS in both strains (Fig. S1 [https://doi.org/10.6084/m9.figshare.22354603]). For instance, PC and PS containing identical FA chains are similarly regulated (compare Fig. 1) graphs and heat maps in Fig. S1 (https://doi.org/10.6084/m9.figshare.22354603). PC and PS containing 16:0 to 20:4, 16:0 to 20:3, 16:1 to 18:1, 16:1 to 20:3, 18:0 to 18:3, 18:1 to 16:1, 18:1 to 18:1, and 18:1 to 18:3 FA, among others, are examples of unsaturated phospholipids whose concentrations significantly increased in the conditional mutant (red asterisks, Fig. S1A and C [https://doi.org/10.6084/m9.figshare.22354603]), possibly explaining the results shown in Fig. 1A. In clear contrast, *xylP*::*hsfA* displayed wild-type levels of unsaturated PE, except for the PE with one or two unsaturations after 30 min of HS (asterisk, Fig. 1C, middle graph). These results are likely caused by the subtle increase in the concentration of phospholipids such as 16:0 to 18:1, 18:1 to 16:0, 18:0 to 18:1, and 18:1 to 18:0 FA (red asterisks, Fig. S1B [https://doi.org/10.6084/m9.figshare.22354603]). Overall, the results show that, differently from PC and PS, the concentration of FA that builds up PE is significantly reduced during the HS in both strains, regardless of the saturation number of the FA. Altogether, these results suggest that *hsfA* depletion affects the levels of mono- and polyunsaturated FA phospholipids, pointing out that HsfA repression exerts a negative global role in unsaturated phospholipid biosynthesis exclusively during HS.

Next, we investigated the role of HsfA during plasma membrane stress. The wild-type and *xylP*::*hsfA* strains were exposed to drugs that disrupt the plasma membrane homeostasis by interfering with sphingolipids, ergosterol, and FA biosynthesis. Given that *xylP*::*hsfA* strains do not grow in the complete absence of xylose, for these experiments, solid MM (1% glucose) was supplemented with 0.06% xylose, a concentration at which the radial growth of the mutant was equivalent to the wild-type strain in the absence of stress, as described elsewhere (36). Interestingly, the *xylP*::*hsfA* mutant was slightly more susceptible to fluconazole and voriconazole than the wild-type strain (Fig. S2 [https://doi.org/10.6084/m9.figshare.22354687]) and significantly more susceptible to AMB, myriocin (an inhibitor of serine palmitoyltransferase that catalyzes the first and rate-limiting step of *de novo* sphingolipid synthesis), and aureobasidin A (an inhibitor of inositol phosphoryl ceramide [IPC] biosynthesis), suggesting that HsfA activity plays a role in the synthesis of constituents of the plasma membrane, especially sphingolipids. In contrast, the *xylP*::*hsfA* mutant exhibited similar susceptibility to the inhibitors of FA biosynthesis (cerulenin and trans-chalcone) compared to the wild-type strain, suggesting that HsfA does not interfere with the FA synthase and the related process of FA elongation (Fig. S2 [https://doi.org/10.6084/m9.figshare.22354687]). Together, these results confirm our lipidomic, suggesting that HsfA is necessary for plasma membrane homeostasis, which is likely associated with the abnormalities in the balance of lipids with different unsaturation levels that are ultimately raw materials to produce complex lipids such as phospholipids and sphingolipids.

### Identification of the A. fumigatus SdeA homolog.

To better understand the increased accumulation of unsaturated phospholipids during HS in the *xylP*::*hsfA* strain (Fig. 1), we investigated the enzymes that desaturate FA molecules in A. fumigatus. In our RNA-seq analysis, we found that FA desaturases (Afu7g05350 and Afu7g05920; Table 1) were downregulated in response to the *hsfA* overexpression, indicating the requirement of this HSF in the biosynthesis of unsaturated FA and the balance of saturated/unsaturated lipids. Enzymes that desaturate the C9 of FA are known as Δ9-fatty acid desaturases, which are highly distributed and conserved from bacteria to mammals. Analysis of A. fumigatus A1163 genome using sequences of S. cerevisiae, Schizosaccharomyces pombe, C. albicans Ole1, A. nidulans, Aspergillus niger, Aspergillus oryzae SdeA, and human stearoyl-CoA desaturases (SCD1 and SCD5) as queries revealed that A. fumigatus A1163 strain possesses two putative Δ9-fatty acid desaturase-encoding genes: AFUB_091500 and AFUB_090930. Consistent with recently reported data (45), the predicted A. fumigatus SdeA protein encoded by the gene AFUB_091500 shows the highest homology with yeast Ole1, the human SCD1, and aspergilli SdeA (Fig. S3 [https://doi.org/10.6084/m9.figshare.22355056]).

A. fumigatus SdeA has the two hallmark domains of membrane-bound desaturases: the FA desaturase and the cytochrome b_5_-like heme domains (Fig. S3 [https://doi.org/10.6084/m9.figshare.22355056]), also demonstrated previously (45). Moreover, SdeA harbors the conserved histidine-box motifs (HXXXXH and HXXHH) inside the FA desaturase domain, which coordinate iron atoms necessary for catalysis (46). S. cerevisiae Ole1 possesses six lysine residues ubiquitinated for proteasome degradation (25, 47), while A. fumigatus SdeA harbors three of these residues (K296, K313, and K379). Interestingly, the human and yeast enzyme sequences have extended N-terminal regions compared to all filamentous fungi sequences. In contrast, fungi uniquely present an extended C-terminal region. Similarly to human SCD1 (46), SdeA has four transmembrane helices that presumably span the endoplasmic reticulum membrane, as determined through the use of DeepTMHMM prediction software (Fig. S3 [https://doi.org/10.6084/m9.figshare.22355056]).

### SdeA is essential and localizes to the endoplasmic reticulum.

To characterize the relevance of SdeA to A. fumigatus biology, we attempted to delete *sdeA* to obtain the Δ*sdeA* strain. Despite many attempts, we obtained no positive transformants (data not shown), suggesting that A. fumigatus *sdeA* is essential for viability. Recently, Wang et al. (45) reported that *sdeA* in A. fumigatus is an essential gene and demonstrated that *sdeA* overexpression affected the vegetative growth and decreased the sensitivity to itraconazole.

**FIG 2:**
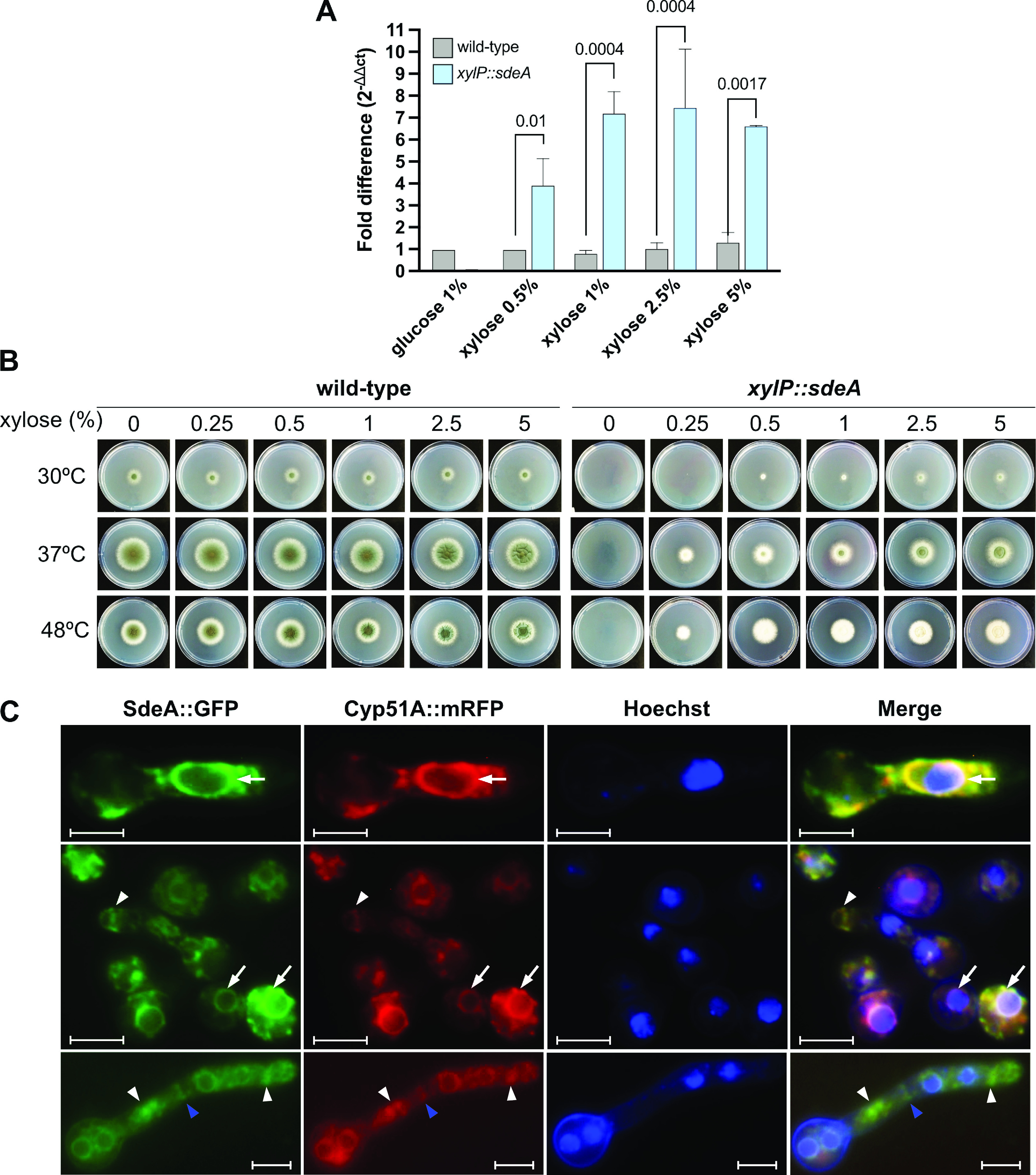
SdeA is essential in A. fumigatus and localizes to the endoplasmic reticulum. (A) *sdeA* expression in the wild-type and *xylP*::*sdeA* strains. The strains were grown for 24 h at 37°C in liquid minimal medium (MM) (1% glucose) supplemented with xylose 1% to allow growth and transferred to fresh MM (1% glucose) or MM supplemented with different concentrations of xylose in the absence of glucose for 4 h to induce *sdeA* expression. The fold increase of each condition represents the normalized values of mRNA in each growth condition relative to the wild-type strain in the control condition (i.e., glucose 1%). Average ± SD (*n* = 3) are shown. The bars indicate statistically significant differences (*P ≤ *0.05; two-way ANOVA and Sidak’s post-test). (B) Radial growth of the wild-type and *xylP*::*sdeA* strains at different temperatures. A total of 1 × 10^4^ conidia of wild-type and *xylP*::*sdeA* strains were inoculated into the center of solid MM plates supplemented with the indicated concentrations of xylose and incubated at 30, 37, or 48°C for 72 h. (C) Fluorescence microscopy was performed with the double-labeled *sdeA*::green fluorescent protein (GFP) *cyp51A*::monomeric red fluorescent protein (mRFP) strain grown in MM (1% glucose) for 8 h at 37°C. On the right, the merged signal of the GFP, mRFP, and Hoechst staining to visualize the nuclei shows that the SdeA::GFP protein accumulates perinuclearly (white arrows), in the septum (blue arrowheads) or distributed throughout the cytosol or subapically (white arrowheads). Magnification, ×100. Bars, 5 μm.

To further assess the functions of *sdeA* in the physiology of A. fumigatus and during the HS, we generate the *xylP*::*sdeA* conditional mutant to study *sdeA* loss-of-function phenotypes (Fig. S4A to C [https://doi.org/10.6084/m9.figshare.22355971]). Similarly to *xylP*::*hsfA*, the expression of *sdeA* in this mutant is under the control of the *xylP* promoter, which is repressed by glucose or induced by xylose (36, 37). To evaluate whether the *xylP* promoter function sharply controls the *sdeA* transcription, the mRNA abundance of *sdeA* in the *xylP*::*sdeA* strain was analyzed by reverse transcription (RT)-qPCR (Fig. 2A). While glucose drastically represses the *sdeA* accumulation in the conditional mutant, increasing xylose concentrations induces *sdeA* expression from 3.5 to 7.0 times. As expected, *sdeA* expression in the wild-type strain remained constant in all xylose concentrations. To evaluate whether *sdeA* repression causes any change in fungal growth, the wild-type and *xylP*::*sdeA* strains were cultured in solid MM supplemented with various xylose concentrations at different temperatures. The conditional mutant was unable to grow in the absence of xylose at all temperatures tested (Fig. 2B), confirming that the A. fumigatus *sdeA* gene is essential, similar to S. cerevisiae and C. albicans *OLE1* (28, 30). A small concentration of xylose (0.25%) was sufficient to induce the growth of the conditional lethal mutant at 37 and 48°C, but not at 30°C. However, the growth of *xylP*::*sdeA* at 37°C did not fully recapitulate that of the wild-type strain, even at higher xylose concentrations. Even at 48°C, a temperature at which *xylP*::*sdeA* mutant grew at equivalent rates to the wild-type strain at high xylose concentrations, the significant reduction in conidiation was not rescued, as noted by the whitish colony color at both 37 and 48°C (Fig. 2B).

Next, we examined the cellular distribution of SdeA in germlings, and for this purpose, a *sdeA*::green fluorescent protein (GFP) strain was constructed (Fig. S4D to F [https://doi.org/10.6084/m9.figshare.22355971]). Phenotypic tests with this strain showed no growth defects compared to the wild-type strain, indicating that such a strain retained significant function of SdeA protein (Fig. S5 [https://doi.org/10.6084/m9.figshare.22356367]). Microscopic inspections of the SdeA::GFP fusion at 30°C showed a strong fluoresce signal around the hyphal nuclei (Fig. 2C). This protein localization pattern is consistent with previous observations of A. fumigatus proteins located in the perinuclear endoplasmic reticulum (48, 49). Moreover, it is also possible to observe intense fluorescence signals at the hyphal tips, as well as subapical and basal regions of the hyphae in an elongated network fashion throughout the cytoplasm and septum, suggesting that part of the SdeA::GFP protein presents peripheral endoplasmic reticulum and septum localization resembling that of Cyp51A (48), which was used to confirm the localization of SdeA to the endoplasmic reticulum (ER) compartments (Fig. 2C).

**FIG 3:**
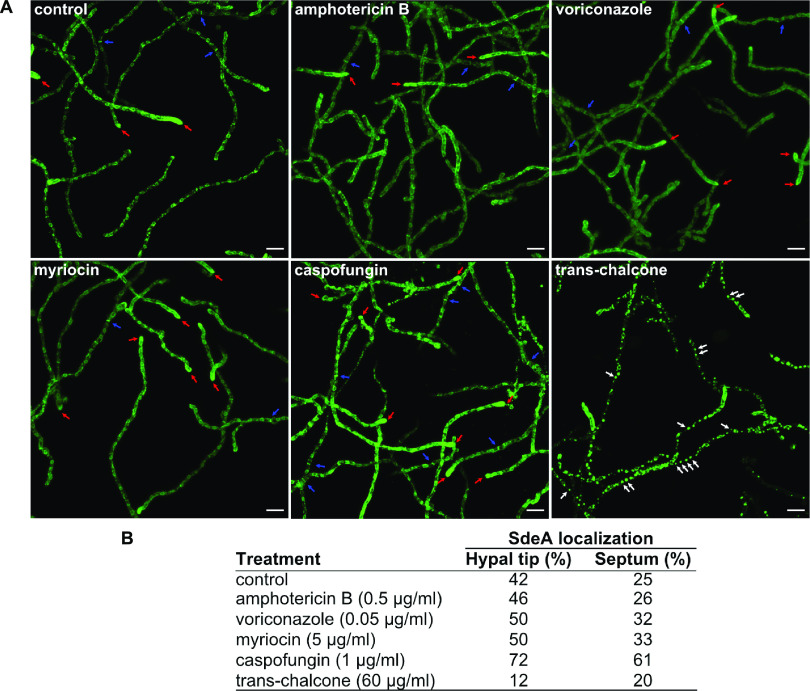
SdeA reallocation in the presence of antifungals. (A) Localization of the SdeA protein was assessed following 1 h of exposure to subinhibitory concentrations of the antifungals amphotericin B (0.5 μg/mL), voriconazole (0.05 μg/mL), myriocin (5 μg/mL), and caspofungin (1 μg/mL) and 20 min to trans-chalcone (60 μg/mL). Fluorescence images represent optical stacks covering the entire hyphae in focus. Magnification, ×40. Bars, 10 μm. Red arrows indicate endoplasmic reticulum (ER) apical localization of SdeA, and blue arrows indicate septa. White arrows indicate punctuated fluorescent structures distributed alongside the hyphae observed exclusively in the trans-chalcone-treated cultures. (B) Quantification of fluorescent apical tips and septa following treatment with the listed antifungals. At least 80 hyphae were analyzed at each condition.

It has been demonstrated that Cyp51A and Cyp51B are redistributed in the cell during the treatment with antifungals or cell wall-damaging agents (48). Given that SdeA and Cyp51A colocalize under basal conditions, we assessed whether the localization of SdeA at the hyphal tip or in the septum would reallocate in the presence of antifungals. We observed that the echinocandin caspofungin (CASP) and the fatty acid synthase inhibitor trans-chalcone significantly caused the most evident reallocation of SdeA (Fig. 3). Quantification demonstrated a significant translocation of SdeA to the hyphal tip of cells treated with CASP, suggesting that monounsaturated FAs are required at the cell membrane to cope with the cell wall damage caused by β-glucan synthase inhibition at the vulnerable sites of apical growth. In the case of trans-chalcone, the ER morphology was dramatically altered, revealing a loss of the ER network associated with the presence of prominent granular fluorescent structures distributed throughout the hyphae, even though septum localization was minimally altered (Fig. 3). It is noteworthy that this abnormal organization of large punctate GFP signals within the cytoplasm is specific to SdeA and not a general response to trans-chalcone since no similar reallocation was observed for the ER-resident protein DapA::GFP challenged by 60 μg/mL of trans-chalcone (Fig. S6 at https://doi.org/10.6084/m9.figshare.22356781). *dapA* encodes a cytochrome *b*_5_-like heme-binding damage resistance protein previously described as involved in ergosterol biosynthesis and azole susceptibility, kindly provided by Ling Liu (Nanjing Normal University, Nanjing, China) (49). In the presence of trans-chalcone, the normal distribution of DapA confirms that the ER structure is not dismantled. These results indicate that SdeA is an essential endoplasmic reticulum-resident protein in A. fumigatus, playing a role in maintaining cell membrane and cell wall organization upon different damaging compounds. Further experimentation is required to investigate how trans-chalcone affects SdeA function.

### SdeA is required for unsaturated FA biosynthesis.

**FIG 4:**
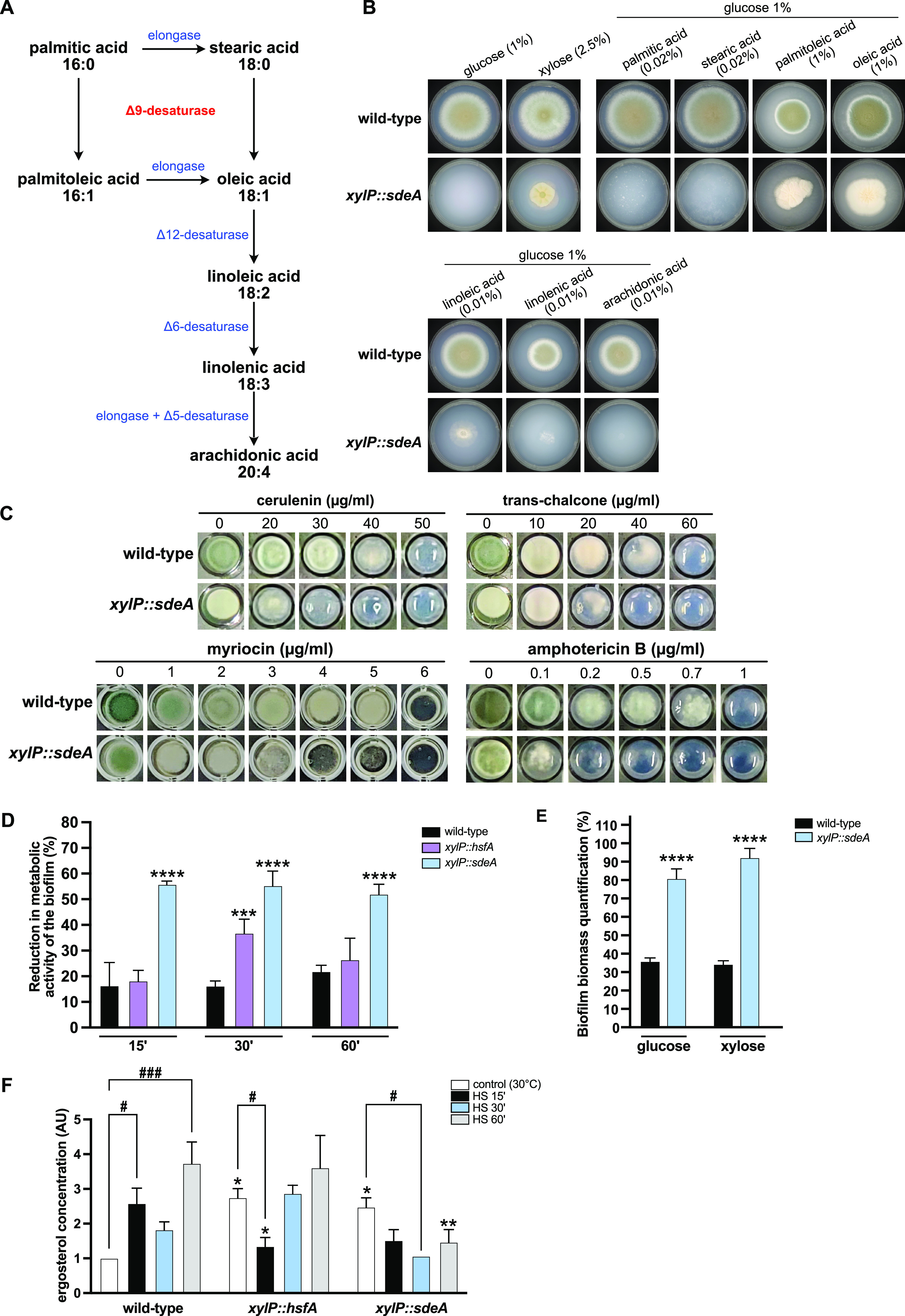
The *xylP*::*sdeA* mutant is auxotrophic for monounsaturated fatty acids and susceptible to plasma membrane stress. (A) Scheme of the fatty acid biosynthetic pathway in fungi indicating the activity of Δ9 desaturase SdeA (red letters). (B) Radial growth of the wild-type and *xylP*::*sdeA* strains in the presence of different concentrations of saturated (palmitic and stearic), monounsaturated (palmitoleic and oleic), and polyunsaturated (linoleic, linolenic, and arachidonic) fatty acids. 1 × 10^4^ conidia of the wild-type and *xylP*::*sdeA* strains were inoculated in solid minimal medium (MM) supplemented with the indicated concentrations of glucose, xylose, and fatty acids and incubated at 37°C for 96 h. (C) Phenotypic assay of the *xylP*::*sdeA* mutant in the presence of plasma membrane stress. A total of 1 × 10^4^ conidia of the wild-type and *xylP*::*sdeA* strains were inoculated in solid MM (1% glucose) supplemented with 2.5% xylose (a concentration at which the radial growth of the mutant was equivalent to the wild-type strain in the absence of stress) and the indicated concentrations of myriocin, amphotericin B, cerulenin, and trans-chalcone. The plates were incubated at 37°C for 72 h. (D) HsfA and SdeA contribute to A. fumigatus caspofungin resistance. The 2,3-bis-(2-methoxy-4-nitro-5-sulfophenyl)-2H-tetrazolium-5-carboxanilide salt (XTT) assay was used to measure the metabolic activity of mature biofilm of wild-type, *xylP*::*hsfA*, and *xylP*::*sdeA* strains. The biofilms were obtained by growing each strain for 18 h in MM (1% glucose) supplemented with 1% xylose at 37°C in 24-well plates. Biofilms were then washed with MM and incubated in MM (glucose 1%; no xylose) for 4 h at 37°C for *xylP* repression. Cell wall stress was induced by incubating repressed biofilms with caspofungin (0.5 μg/mL) for 15, 30, or 60 min. The results were expressed as means ± SD (*n* = 3). ***, *P ≤ *0.001; ****, *P* ≤ 0.0001 (two-way ANOVA and Sidak’s post-test). (E) Biofilm biomass was evaluated by crystal violet absorbance at 570 nm and expressed as the percentage of biomass adhesion considering 100% for the wild-type strain. The biofilms were obtained by growing each strain for 20 h in MM (1% glucose) supplemented with 1% xylose at 37°C. The biofilms were then washed with prewarmed MM and further incubated in MM 1% glucose (no xylose) or MM 1% xylose (no glucose) for 4 h at 37°C for *xylP* repression or induction, respectively. The results were expressed as means ± SD (*n* = 12). ****, *P ≤ *0.001 (two-way ANOVA and Sidak’s post-test). (F) Ergosterol quantification. The strains were grown in liquid MM (1% glucose) supplemented with xylose 1% for 24 h at 30°C to allow growth. Subsequently, the mycelia were washed twice with MM and incubated for 4 h at 30°C in MM (glucose 1%; no xylose) for *xylP* repression. Heat shock (HS) was induced by transferring the mycelia to fresh preheated MM (glucose 1%; no xylose) for an additional 15, 30, and 60 min of incubation at 48°C. The control was left at 30°C. Ergosterol was extracted and quantified. The results were expressed as means ± SD (*n* = 3). *, *P ≤ *0.02; **, *P ≤ *0.002 (two-way ANOVA and Sidak’s post-test relative to wild-type in the same condition). The bars (#) indicate a statistically significant difference: #, *P ≤ *0.03; ###, *P ≤ *0.0005 (two-way ANOVA and Sidak’s post-test).

To investigate the role of SdeA in FA biosynthesis and identify the potential substrates and products of this enzyme, a phenotypic assay for the *xylP*::*sdeA* strain was performed using different FA supplementations. The FA biosynthesis pathway in fungi is shown in Fig. 4A, emphasizing the requirement of the Δ9-desaturase enzyme in the generation of the Δ9 monounsaturated FA palmitoleic and oleic acids, which are precursors of polyunsaturated fatty acids (PUFAs). As expected, the repressed *xylP*::*sdeA* mutant (glucose 1%; no xylose) could not grow when supplemented by saturated FA such as palmitic and stearic acids as the sole source of lipids (Fig. 4B). In contrast, the growth of the conditional lethal mutant was restored in the presence of monounsaturated FAs, palmitoleic acids, and oleic acids under the same growth conditions (glucose 1%; no xylose). These results indicate that *xylP*::*sdeA* mutant is auxotrophic for monounsaturated FA since the addition of palmitoleic and oleic acids bypasses the *sdeA* requirement for the synthesis of Δ9-monounsaturated FA. It is noteworthy that the conidiation defect observed in this mutant was not fully rescued by the supplementation with such molecules, suggesting that endogenous production of oleic and palmitoleic acid is essential for the A. fumigatus conidiation. The supplementation of PUFAs such as linoleic, linolenic, and arachidonic acids accounted only for poor or no growth of the *xylP*::*sdeA* strain (Fig. 4B), reinforcing that the main products of the SdeA enzyme are monounsaturated FA. In addition, higher concentrations of all FA used here inhibited the growth of both strains (data not shown). These results confirm that SdeA is essential for synthesizing monounsaturated FA.

To further determine whether *sdeA* had broad physiological impacts on A. fumigatus cell membrane homeostasis, we grew the *xylP*::*sdeA* lethal conditional mutant in the presence of cell membrane-disturbing agents and different inhibitors of lipid biosynthesis. To allow growth of the *xylP*::*sdeA* mutant in this experiment, the xylose concentration of 2.5% was chosen, since radial growth was nearly similar to the wild type (Fig. 2B). Interestingly, *xylP*::*sdeA* strain was as sensitive as the wild type to SDS and voriconazole or fluconazole (data not shown). In contrast, the conditional mutant exhibited increased sensitivity to AMB and myriocin (Fig. 4C). Moreover, the FA biosynthesis inhibitors cerulenin and trans-chalcone significantly inhibited the growth of the *xylP*::*sdeA* mutant compared to the wild-type strain (Fig. 4C). These results highlight the importance of SdeA in FA metabolism with implications for complex lipids such as sphingolipids.

To probe whether the altered balance of saturated/unsaturated FA translated into possible cross talk between SdeA activity and CWI, we investigated the sensitivity of mature A. fumigatus biofilm of the wild-type and *xylP*::*sdeA* strains to CASP. Given the involvement of HsfA in the increased accumulation of PC and PS (Fig. 1) and the CWI maintenance (36), the *xylP*::*hsfA* strain was also included in this experiment. The 2,3-bis-(2-methoxy-4-nitro-5-sulfophenyl)-2H-tetrazolium-5-carboxanilide salt (XTT) assay was employed as previously described (19). The *xylP*::*sdeA* mutant grown under repressive (glucose 1%, no xylose) conditions exhibited a significant decrease in metabolic activity (55%) when the biofilm was treated with CASP compared to the wild type, regardless of the exposure time (Fig. 4D). Surprisingly, the *xylP*::*sdeA* mutant showed an enhanced biomass accumulation on the biofilms compared to the wild-type strain (Fig. 4E), yet the hyphae of such a strain displayed no significant alterations in cell wall thickness when inspected by transmission electron microscopy (data not shown). Notably, the *xylP*::*sdeA* strain susceptibility to CASP or other cell wall-disturbing agents (e.g., calcofluor white, Congo red, and caffeine) was equivalent to the wild-type strain in radial growth assays of conidia on agar plates (data not shown). Hence, while *sdeA* loss of function causes no impact on conidia tolerance to cell wall stress, depletion of *sdeA* significantly sensitizes A. fumigatus biofilm to CASP treatment. Our results highlight a novel promising synergistic effect of β-glucan inhibition via echinocandin antifungals and depletion of monounsaturated FA in the cell membrane of A. fumigatus biofilm.

Interestingly, despite the documented susceptibility of *xylP*::*hsfA* conidia to CASP and the abnormalities in the CWI upon *hsfA* depletion in fungal biofilms (36), this effect was much lower in the *xylP*::*hsfA* strain (35% reduction) and significant only after 30 min of CASP exposure (Fig. 4D). Aiming to understand further the membrane perturbations imposed by the repression of *hsfA* and *sdeA*, we measured the ergosterol content in the conditional mutants under control (30°C) and HS conditions as an additional approach to assessing the membrane composition of the mutants. We observed that under control conditions, there is a significant increase in the ergosterol levels in both repressed conditional mutants (2.5-fold) compared to the wild-type strain (Fig. 4F). Such an increase can be an initial explanation for the lack of increased susceptibility to azoles presented by these strains in the phenotypic tests (data not shown and Fig. S2 [https://doi.org/10.6084/m9.figshare.22354687]). While the HS increased ergosterol content after 15 and 60 min in the wild-type strain, the same was not observed in the *xylP*::*hsfA* or *xylP*::*sdeA* mutants. In fact, significant decreases were recorded after 15 or 30 min of HS in the *xylP*::*hsfA* and *xylP*::*sdeA* strains, respectively. At least for *xylp*::*sdeA* mutant strain, these results highlight that ergosterol content reflects the opposite pattern of accumulation recorded for the wild-type strain, which may be a consequence of the altered ratio of unsaturated to saturated fatty acids.

**FIG 5:**
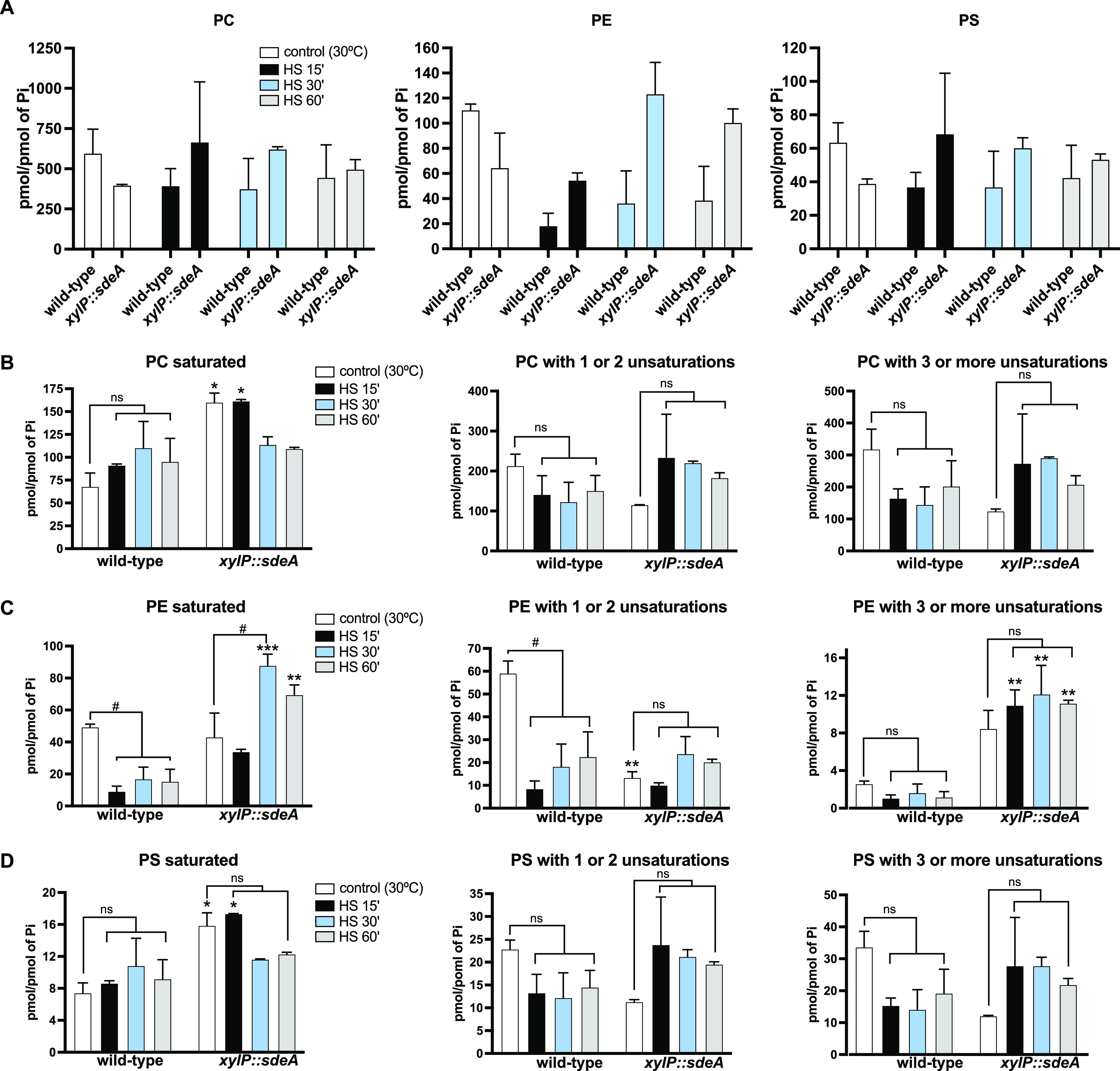
SdeA is required for unsaturated fatty acid biosynthesis. (A) General proportion of the phospholipids classes: phosphatidylcholine (PC), phosphatidylethanolamine (PE), and phosphatidylserine (PS) measured in the wild-type and *xylP*::*sdeA* strains. The strains were grown in liquid minimal medium (MM) (1% glucose) supplemented with xylose 1% for 24 h at 30°C to allow growth. Subsequently, the mycelia were washed twice with MM and incubated for 4 h at 30°C in MM (glucose 1%; no xylose) for *xylP* repression. Heat shock (HS) was induced by transferring the mycelia to fresh preheated MM (glucose 1%; no xylose) for an additional 15, 30, and 60 min of incubation at 48°C. The control was left at 30°C. (B to D) Levels of the phospholipids PC (B), PE (C), and PS (D) grouped according to the degree of saturation of the fatty acid chains: saturated lipids, lipids with one or two unsaturations, and lipids with three or more unsaturations. Lipids were measured by mass spectrometry, and the values were normalized by the inorganic phosphate (P_i_) levels. *, *P ≤ *0.05; **, *P ≤ *0.01; and ***, *P ≤ *0.001 (two-way ANOVA and Sidak’s post-test in relation to wild-type in the same condition). Bars superscripted with # (*P ≤ *0.05) indicate significant comparisons within the same strain to evidence the HS effect.

Next, to explore how *sdeA* affects unsaturated FA and phospholipids in A. fumigatus, we again quantified PC, PE, and PS levels in the repressed *xylP*::*sdeA* mutant. To this end, the wild-type and *xylP*::*sdeA* strains were grown in liquid MM (1% glucose) supplemented with xylose 1% for 24 h at 30°C. Subsequently, the mycelia were washed twice with MM and incubated for 4 h at 30°C in MM (glucose 1%; no xylose) for *xylP* repression. HS was induced by transferring the mycelia to fresh preheated MM (48°C) for 15, 30, and 60 min of incubation at 48°C. The control was left at 30°C. Our results demonstrated that the depletion of *sdeA* did not significantly alter the accumulation of these phospholipids compared to the wild-type strain (Fig. 5A). When the phospholipids species were grouped by the degree of saturation, the results confirmed that the abundance of saturated PC, PE, and PS was significantly higher in the *xylP*::*sdeA* mutant compared to the wild-type strain. Interestingly, the higher accumulation of saturated PC and PS in the *xylP*::*sdeA* mutant was detected at 30°C and after 15 min of HS. For PE, significant increases were observed later, i.e., after 30 and 60 min of HS (asterisks in Fig. 5B to D). The individual quantification of PC, PE, and PS species in the *xylP*::*sdeA* strain demonstrated that the main saturated FAs associated with these phospholipids comprise 16:0 to 16:0, 16:0 to 18:0, 18:0 to 16:0, and 18:0 to 18:0 (purple asterisks in Fig. S1A to C) ([https://doi.org/10.6084/m9.figshare.22354603]). This effect is highly consistent with the function of SdeA in converting saturated FA into monounsaturated FA, indicating that the *sdeA* depletion causes the accumulation of SdeA substrates and, consequently, saturated phospholipids. Accordingly, the *xylP*::*sdeA* mutant synthesized less unsaturated PE with one or two unsaturations than the wild-type strain at 30°C (asterisks, Fig. 5C, middle graph). Nevertheless, the levels of unsaturated PC and PS did not significantly change.

Surprisingly, the concentration of PE with three or more unsaturations remarkably increased in the *xylP*::*sdeA* mutant (~2.8-fold) at both basal and HS conditions compared to the wild-type strain (asterisks, Fig. 5C, right graph), although *sdeA* repression significantly increased the levels of saturated PE (asterisks, Fig. 5C, left graph). These data suggest the participation of another uncharacterized FA desaturase that preferentially desaturates PE containing PUFA in its structure. Examples of phospholipids with three or more unsaturations that increased in the *xylP*::*sdeA* mutant are 16:0 to 18:3 and 18:0 to 18:3 (green asterisks, Fig. S1B [https://doi.org/10.6084/m9.figshare.22354603]).

### Sphingolipids levels are affected by *hsfA* but not *sdeA* depletion.

The basic structure of sphingolipids harbors two FA molecules, which present an abundant chemical diversity regarding the length of the carbon chain and the number of unsaturation (50). Therefore, the activity of FA desaturases is essential to generate the pool of the different classes of sphingolipids that will be further incorporated into the plasma membrane.

To determine whether *hsfA* or *sdeA* affects the metabolism of sphingolipids and chemically defines how saturated and unsaturated FAs decorating these molecules are being altered by the depletion of these genes, the wild-type, *xylP*::*hsfA*, and *xylP*::*sdeA* strains were submitted to a sphingolipidomic analysis under the same growth conditions used above. We observed that the HS had no significant impact on the accumulation of total sphingolipids in the wild-type and *xylP*::*sdeA* strains (Fig. S7A [https://doi.org/10.6084/m9.figshare.22357045]). However, the *xylP*::*hsfA* mutant presented a significant increase in the abundance of total sphingolipids after 15 and 30 min of HS, suggesting that HsfA regulates sphingolipid biosynthesis (Fig. S7A [https://doi.org/10.6084/m9.figshare.22357045]). In fact, the major increases among the sphingolipids classes for the *xylP*::*hsfA* mutant were recorded in the group of glucosyl ceramides (GlcCers; ~4.5-fold), and phytoceramides (PCers; ~2.2-fold), while no variation was observed for OH-Cer and OH-PCer molecules (asterisks, Fig. S7B [https://doi.org/10.6084/m9.figshare.22357045]). We observed increases in the concentration of sphingolipids containing FAs with a variable number of carbons and unsaturations belonging to the group of dihydroceramides (DHCs), ceramide (Cer), PCer, GlcCer, and IPC (blue asterisks, Fig. S7C [https://doi.org/10.6084/m9.figshare.22357045]). Differently from wild-type yeast that accumulates sphingolipids during HS (51), it is possible that significant increases in the A. fumigatus total sphingolipid content were achieved only when thermotolerant growth was severely impaired in the *xylP*::*hsfA* mutant and not in the wild type. Our results reinforce the importance of sphingolipids in HS adaptation.

Although our results support the idea that *sdeA* repression is not as crucial for sphingolipid biosynthesis as for phospholipid biosynthesis, we observed that *sdeA* repression led to a higher accumulation of some DHC, PCer, and IPC, compared to the wild-type strain (red asterisks; Fig. S7C [https://doi.org/10.6084/m9.figshare.22357045]). It is noteworthy that all these species have in common saturated FA chains both in the LCB and the amide-linked FA, reinforcing that *sdeA* repression causes not only accumulation of saturated phospholipids (Fig. 4) but also saturated sphingolipid species (Fig. S7C to D [https://doi.org/10.6084/m9.figshare.22357045]).

### *hsfA* depletion compromises SdeA expression.

**FIG 6:**
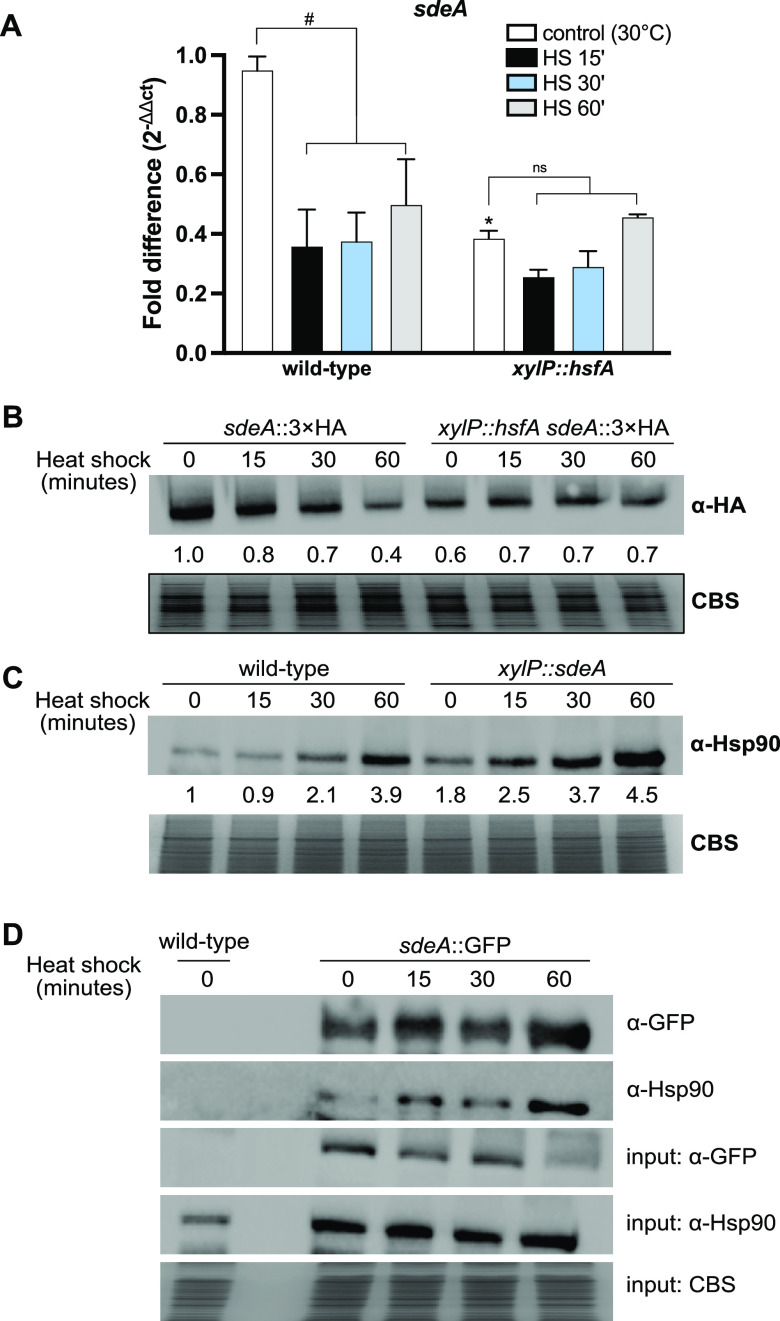
HsfA regulates SdeA expression and SdeA physically interacts with Hsp90. (A) The *sdeA* mRNA levels are decreased in the *xylP*::*hsfA* strain during *hsfA* repression. The strains were grown in liquid minimal medium (MM) (1% glucose) supplemented with xylose 1% for 24 h at 30°C to allow growth. Subsequently, the mycelia were washed twice with MM and incubated for 4 h at 30°C in MM (glucose 1%; no xylose) for *xylP* repression. Heat shock (HS) was induced by transferring the mycelia to fresh preheated MM (glucose 1%; no xylose) for an additional 15, 30, and 60 min of incubation at 48°C. The control was left at 30°C. Total mRNA was evaluated by RT-qPCR and normalized by the β-tubulin gene. The data are from three experimental and independent replicates (average ± SD). The fold difference for each condition represents the total mRNA normalized to the wild-type control. The bars indicate statistically significant differences: #, *P ≤ *0.01 (two-way ANOVA, with Sidak’s post-test). *, *P ≤ *0.01, a significant comparison between the wild-type and *xylP*::*hsfA* strains in the same time point. (B) SdeA protein expression is repressed in the *xylP*::*hsfA* genetic background during *hsfA* repression. Samples were obtained as described above for panel A. α-HA antibody was used to recognize the SdeA::3×HA protein in Western blot analysis of *sdeA*::3×HA and *xylP*::*hsfA sdeA*::3×HA strains during HS. (C) Hsp90 protein expression is induced in the *xylP*::*sdeA* genetic background during *sdeA* repression. The samples were obtained as described above for panel A. α-Hsp90 antibody was used to recognize the Hsp90 protein in Western blot analysis of wild-type and *xylP*::*sdeA* strains during HS. (D) SdeA and Hsp90 physically interact *in vivo*. Wild-type and *sdeA*::GFP strains were grown in MM (1% glucose) for 24 h at 30°C and subjected to HS as described for panel A. The interaction between the proteins was verified through coimmunoprecipitation using GFP-Trap-agarose resin and α-GFP, which detects the SdeA::GFP protein, and α-Hsp90 antibodies. For all Western blot analysis, the expression values are arbitrary and calculated by densitometry using the ImageJ software (85). Coomassie blue staining (CBS) was used as the loading control. HA, hemagglutinin.

Given that SdeA activity is tightly coordinated with temperature increase to adjust membrane composition and considering that repression of *hsfA* caused an increase in mono- and polyunsaturated PC and PS during the HS (Fig. 1), we asked whether the expression of SdeA is influenced by HsfA. We initially assessed the *sdeA* mRNA levels in the *xylP*::*hsfA* mutant under repressive conditions (glucose 1%; no xylose). The *sdeA* gene expression in the control condition was ~50% lower in the conditional mutant in comparison to the wild-type strain (Fig. 6A), suggesting that HsfA has a positive impact on the basal expression of *sdeA*. Consistent with the activity of a Δ9-desaturase, the *sdeA* mRNA abundance significantly decreased during the HS in the wild-type strain, while the same did not occur in the repressed *xylP*::*hsfA* under the HS condition.

Next, SdeA protein levels were evaluated under the depletion of *hsfA* (glucose 1%; no xylose), by submitting the *xylP*::*hsfA sdeA*::3× hemagglutinin (HA) and *sdeA*::3×HA strains to a Western blot analysis. The construction of these mutants is reported in Fig. S4G to I ([https://doi.org/10.6084/m9.figshare.22355971]). Both strains showed no growth defects compared to parental strains (Fig. S5B [https://doi.org/10.6084/m9.figshare.22355971]), indicating that the introduction of 3×HA epitope retains significant function of SdeA protein. Consistent with the mRNA accumulation profile, SdeA protein levels were significantly reduced (40%) in the *xylP*::*hsfA* strain pre-HS, again suggesting a positive role of HsfA over SdeA expression at basal (30°C) condition (Fig. 6B). Interestingly, SdeA protein levels were maintained at low and sustained levels throughout the HS in the *xylP*::*hsfA* strain, whereas SdeA levels in the wild-type strain dropped significantly (20%, 30%, and 60% after 15, 30, and 60 min, respectively). These data further support a positive role for HsfA over SdeA expression only under basal conditions. We also interpret these data to suggest that *hsfA* repression leverages the level of the desaturase, thus impairing the natural drop in *sdeA* mRNA and protein levels during HS, which is clearly observed in the wild-type strain. As a result and guided by the impaired *hsfA* repression during the HS, the *xylp*::*hsfA* strain grown under repressive conditions subjected to HS experiences increased accumulation of unsaturated phospholipids and sphingolipids recorded in Fig. 1 and Fig. S7 (at https://doi.org/10.6084/m9.figshare.22357045), respectively.

Prior research has hinted at the importance of FA metabolism, HSP activation, and HS response in other human fungal pathogens (34, 35). Notably, *OLE1^sdeA^* depletion in C. albicans prevented complete activation of Hsf1^HsfA^, which downregulated HSP expression upon HS (24). As *hsfA* and the HSP *hsp90* expression are coordinated during the HS (36), we asked whether SdeA influences Hsp90 protein expression, the main transcriptional target of HsfA (36). We observed that under *sdeA* depletion (glucose 1%; no xylose), Hsp90 protein levels were increased in the *xylP*::*sdeA* mutant compared to the wild-type strain at all time points: 80% under control conditions and 178%, 76%, and 15% after 15, 30, and 60 min of HS, respectively (Fig. 6C). Given its broad chaperone activity, Hsp90 has an ever-growing list of client proteins favored by various physiological or stressful conditions to assist the activity of the target protein. To verify whether SdeA is part of the Hsp90 clientele *in vivo*, a coimmunoprecipitation (co-IP) assay was performed using the GFP-Trap resin and the *sdeA*::GFP strain. We discovered that SdeA and Hsp90 physically interacted under basal and HS conditions (Fig. 6D). Altogether, our results suggest that the absence of SdeA is sufficient to induce HS response and disclose a relevant connection between SdeA and Hsp90 in A. fumigatus.

## DISCUSSION

Recently, we have shown that HsfA plays a critical role in the HS response and the cell signaling funneling into the CWI pathway to govern cell wall integrity and thermotolerance (19, 36). Here, we demonstrate that HsfA negatively regulates the biosynthesis of phospholipids, sphingolipids, and unsaturated FAs that shape these molecules. The main biochemical findings supporting these assumptions are the significant increases in the content of PC and PS during HS in the *xylP*::*hsfA* mutant. In addition, unsaturated FA present in PC and PS were significantly increased in the mutant, whereas minor changes in the composition of unsaturated FA were observed for the PE. Although PE is the second most abundant phospholipid in mammalian and fungal cells, it is the only class of phospholipids whose concentration significantly dropped in both strains during the HS, suggesting that unlike PC and PS, PE is not regulated by *hsfA*. While PC is mostly found in the outer leaflet of the cell, PE and PS are located in the inner (cytoplasmic) leaflet of the lipid bilayer (52). PE is particularly enriched in the mitochondrial inner membranes of mammalian cells, where it is produced *in situ* via decarboxylation of serine moiety of PS (53). It is possible that the ethanolamine supply during the HS is perturbed, hampering the accumulation of PE catalyzed by the CDP-ethanolamine pathway, the only pathway that synthesizes *de novo* PE in eukaryotes. Curiously, little is known about the origin of the ethanolamine that feeds this pathway in mammals (54), while virtually no information is available in fungi, although PS and PE can act as modulators of virulence in C. albicans (reviewed in reference 55).

The addition of double bonds into the hydrocarbon chains of FA is accomplished by desaturases. A. fumigatus possess eight putative FA desaturases previously identified *in silico* (56). We originally found *sdeA* by investigating downregulated genes during *hsfA* overexpression in this study (Table 1) and previously in data sets associated with *hsfA* depletion and HS series (36). Given the results presented here, as well as others, we demonstrated that SdeA is the closest homolog of S. cerevisiae and C. albicans *OLE1* and A. nidulans *sdeA* (28, 30, 31, 45). Both genetics and lipidomics analysis and the localization of SdeA at the endoplasmic reticulum suggest that *sdeA* is the main desaturase of palmitic and stearic FA in A. fumigatus. Thus, repression of *xylP*::*sdeA* increases the amount of saturated FA present in PC and PS and some species of sphingolipids, accompanied by a decrease in unsaturated FA enrichment in such molecules. Consistently, A. fumigatus *sdeA* overexpression leads to the overall accumulation of unsaturated FA, while the opposite was recorded for saturated FA (45).

We found that *sdeA* is involved in conidiation. Likewise, deletion of A. nidulans *sdeA* and depletion of C. albicans *OLE1*, respectively, reduced conidiation or caused defects in the formation of chlamydospores (30, 31). Precedent exists indicating the requirement of FA for bona fide conidiation in aspergilli. For instance, in A. niger, oleic acid represents 31.4% of conidial FA (57), while the conversion of oleic to linoleic acid by the oleate Δ12-desaturase (repressed 3.5 log-fold in our RNA-seq data set; Table 1) also reduced conidiation and mycelial growth in A. nidulans (58).

A most unexpected observation in this study was the significant and specific increase in PE harboring FA with three or more unsaturations during the HS, which presumably hinted at the activity of another Δ9-fatty acid desaturase (Fig. 5C). Intriguingly, when we searched for other desaturase-encoding genes across Af293 and A1163 genomes using *sdeA* nucleotide sequences as queries, the open reading frame (ORF) encoded by Afu7g05350/AFUB_090930, annotated as a putative Δ9-fatty acid desaturase, returned, respectively, in such strains (73% and 69% identity). Given that this gene was not previously identified in the study by Tang et al. (56), we investigated the AFUB_090930 gene by generating a deletion mutant. The AFUB_090930 null mutant showed equivalent growth as the wild-type and reconstituted strains at 30, 37, and 48°C, as well as in the presence of all cell membrane-perturbing agents used in this study (data not shown). These results suggest that AFUB_090930 does not play a role in the A. fumigatus thermotolerant response or membrane stress in A1163 strain. Further inspections in FungiDB (https://fungidb.org/fungidb/app) indicated that AFUB_090930 is a pseudogene with a coding sequence. An in-depth analysis of reads mapping of published transcriptomics data sets and our RNA-seq data (File S1 [https://doi.org/10.6084/m9.figshare.22354441]) consistently showed that AFUB_090930 is transcribed. However, there is no evidence that this transcript is translated into an active desaturase that could explain our results. In fact, we failed to produce translational fusions of AFUB_090930 with GFP or 3×HA epitope (data not shown). Curiously, the ortholog Afu7g05350 identified in the Af293 strain is annotated as a functional gene. Since Afu7g05350 has not been characterized so far, it is unclear whether it encodes a FA desaturase and whether A. fumigatus expresses two functional Δ9-fatty desaturases during the HS. In A. nidulans, two redundant Δ9-desaturases (*sdeA* and *sdeB*) affect FA metabolism (31). Why the synthesis of PE with three or more unsaturations increases under depletion of *sdeA* is an open question. While this may likely be necessary to correct the saturated/unsaturated FA balance in *xylP*::*sdeA* mutant, further investigation into the function of Afu7g05350/AFUB_090930 in the Af293 and A163 strains will be essential to understand whether such genes are functional in these two clinical isolates and to define the mechanism of regulation associated with potential genetic heterogeneity.

Disbalance in the content of FA caused by genetic manipulation or exogenous supplementation of FA is known to affect growth and drug tolerance in pathogenic yeasts (23, 59, 60). Conidia of the repressed *xylP*::*sdeA* mutant were significantly susceptible to the inhibition of FA synthesis and to AMB, although not to azoles (data not shown). Interestingly, the exogenous supplementation of oleic acid and the PUFAs linoleic acid and linolenic acid increased the resistance of A. fumigatus conidia to itraconazole, but not to voriconazole or CASP (45). Here, we observed that the lack of CASP susceptibility of the *xylP*::*sdeA* conidia was not directly transferable to the biofilm state of such strain challenged with this drug. Instead, the marked reduction in the ability of the *xylP*::*sdeA* biofilms to reduce XTT following CASP treatment strongly suggests that depletion of endogenous monounsaturated FA, the concomitant accumulation of saturated FA, and the associated alterations in ergosterol accumulation enhanced the sensitivity to β-glucan inhibition (Fig. 4). Consistently, CASP significantly increased the SdeA apical tip localization, reinforcing previous observations that reallocation of ER proteins such as SdeA, Cyp51A, and Cyp51B at the hyphal tip after exposure to antifungals may represent a general A. fumigatus response to stresses that compromise the apical growth (48). Additional experimentation is needed to reveal the connections between saturated/unsaturated FA balance, ergosterol content, and cell wall integrity perturbation in A. fumigatus biofilm. Aside from some likely alterations in the cell permeability, which may account for different levels of drug uptake, our results point out a potential synergy between echinocandins and inhibition of monounsaturated FA synthesis, which specifically increases the efficacy of this antifungal against the biofilm state found in established infection of most filamentous fungi. Examples of molecules and determinants of virulence that sustain this concept are increasingly relevant in A. fumigatus (61, 62). We also demonstrated that inhibiting FA synthase strikingly altered the localization pattern of SdeA, since the granular structures following trans-chalcone treatment are specific to SdeA and significantly large to be considered stress granules observed in aspergilli (63). We observed an increased accumulation of saturated FA upon *sdeA* depletion, which presumably caused an increase in palmitic and stearic acid content. In A. nidulans, stearic acid accumulation induced by *sdeA* deletion stimulates fatty acid synthesis *via* the upregulation of the *fasA* gene that encodes the α-chain of FA synthase (31). It is possible that the chemical imbibition of FA synthase by trans-chalcone deteriorates the pool of saturated FA in the *sdeA*-depleted cells, and one of the consequences is the SdeA reallocation from the ER.

**FIG 7:**
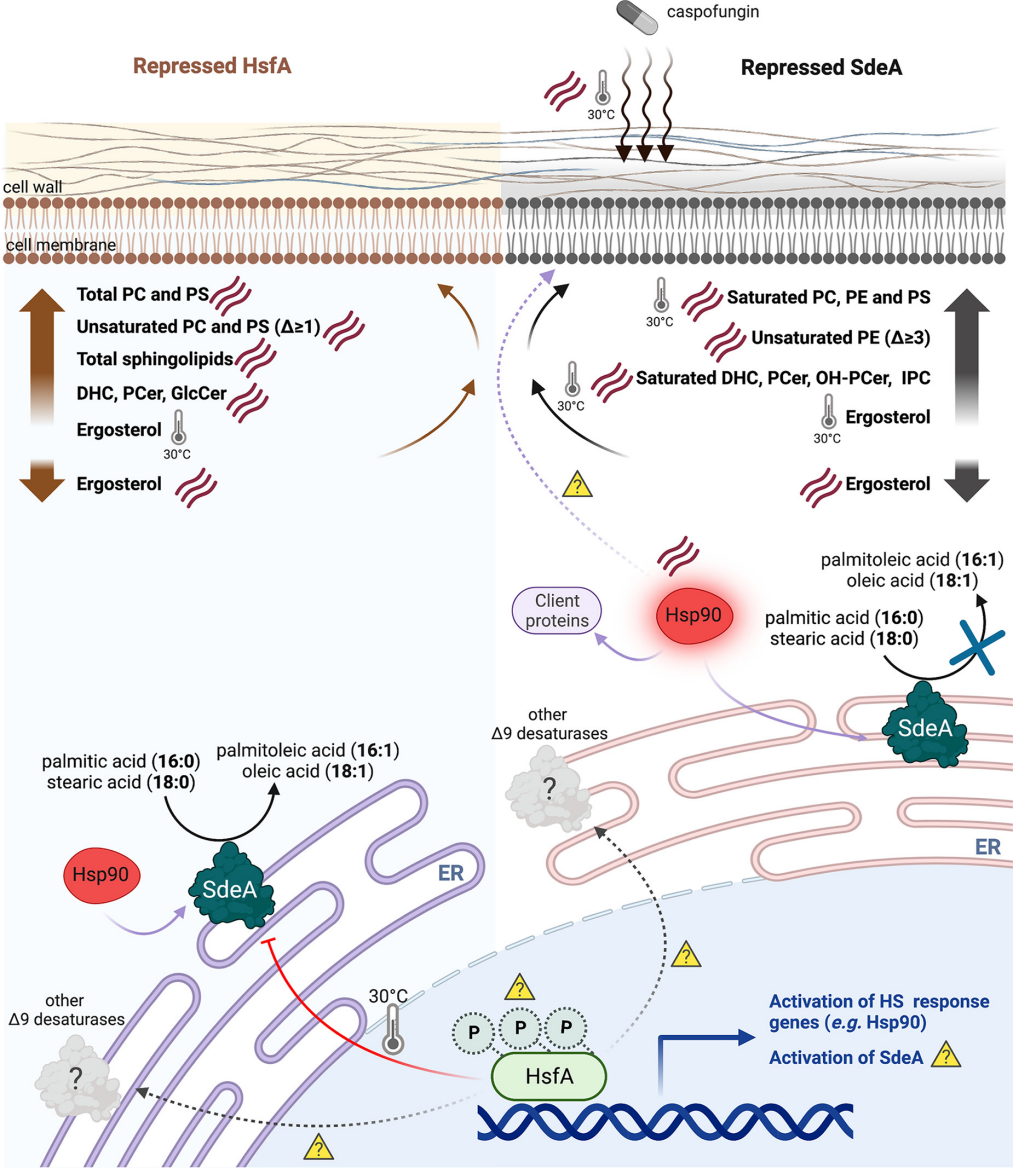
The interplay cross talk between HsfA, SdeA and membrane lipids metabolism under basal conditions and heat shock (HS) stress. Under heat stress (indicated by the three parallel lines), HsfA is induced and activated, triggering the expression of genes related to HS response, including *hsp90* and possible *sdeA* (question marks). The phosphorylation status of *hsfA* under the conditions employed here is unknown. Repression of *hsfA* (left panel) under basal conditions (30°C), but not under HS, downregulates *sdeA* mRNA and protein levels (red arrow), likely lowering the activity of SdeA. Under HS, normal levels of SdeA are present in the *hsfA*-repressed cells, resulting in increased levels of total phospholipids, including unsaturated PC(phosphatidylcholine) and PS(phosphatidylserine) in the plasma membrane, concomitantly to an increase in the pool of sphingolipids, including dihydroceramides (DHCs), phytoceramides (PCers), and glucosyl ceramides (GlcCers) and a drop in ergosterol levels. It is unknown whether A. fumigatus encodes other Δ9 desaturases (see Discussion for details) that support the increased levels of unsaturated lipids and whether they are controlled by *hsfA* (question marks). Hsp90 is upregulated at basal and HS conditions during the repression of *sdeA* (right panel). SdeA physically interacts with Hsp90, the main transcriptional target of HsfA also under basal and HS conditions. The direct function of Hsp90 over lipids at the cell membrane is unknown (purple dotted line and question mark). Repression of *sdeA* during the HS caused an increased accumulation of all classes of saturated phospholipids and some saturated species of sphingolipids accompanied by a decrease in ergosterol content. The altered composition of cell membrane lipids in the depleted *sdeA* cells synergizes the action of caspofungin over mature biofilms in which *sdeA* was depleted under both basal (30°) and HS conditions, thus decreasing cell viability. Created with BioRender.com. ER, endoplasmic reticulum; DHC, dihydroceramide; GlcCer, glucosylceramide; PCer, phytoceramide; OH-PCer, hydroxyphytoceramide; IPC, inositol phosphoryl ceramide.

Previous experiments indicated that *OLE1* depletion prevents the complete activation and phosphorylation of Hsf1 in C. albicans, which decreases mRNA levels of HSPs such as *HSP104* and *HSP21*, while cells retained constant levels of Hsp90 protein expression (24). Although HsfA activation was not studied in our work (Fig. 7), we discovered that Hsp90 protein levels were significantly induced under depletion of *sdeA*. Interestingly, neither *hsfA* nor *hsp90* were modulated in the RNA-seq analysis upon overexpression of A. fumigatus *sdeA* (45). Thus, our findings suggest a different regulatory mechanism observed in C. albicans. It has been found in several models that the HSPs levels can be modulated as a result of changes in the plasma membrane, without the occurrence of HS or protein denaturation (34, 35, 64–67). Different HSPs are associated with the plasma membrane in specific lipid microdomains, which may be a strategy for organisms to compartmentalize HSPs close to other signaling proteins that respond to cell surface receptors (68, 69). For instance, a direct strong interaction between Hsp90 and different compositions of lipids was reported. Hsp90 preferentially binds to more unsaturated phospholipid species, and the affinity was higher with negatively charged lipids than with zwitterionic lipids (70). We propose that the predicted accumulation of saturated FA or the deficiency of some unsaturated fatty acid that build up phospholipids and sphingolipids caused by *sdeA* depletion possibly requires the Hsp90 chaperone activity at higher levels to cope with membrane stress caused by the unbalanced saturated/unsaturated FA ratio in organelles. It is tempting to speculate that the thermophilic nature of A. fumigatus supports this observation; however, the definition of HsfA activation status in the *sdeA*-depleted cells is crucial to validate this concept. Indeed, we observed that depletion of HsfA decreases SdeA levels at non-HS conditions, suggesting that HsfA is necessary to sustain the production of unsaturated FA. In summary, our results highlight the complex interaction between HS and thermophily in A. fumigatus with effector proteins such as HsfA and SdeA and the consequences to the unsaturated/saturated FA in complex lipids such as phospholipids and sphingolipids.

## MATERIALS AND METHODS

### Strains and culture conditions.

The A. fumigatus strains used in this study are described in Table S1 (https://doi.org/10.6084/m9.figshare.22354573). The strains were maintained in complete medium (YG; 2% glucose [wt/wt], 0.5% yeast extract [wt/wt], 1× trace elements) or MM (1% glucose [wt/wt], 1× high-nitrate salts, 1× trace elements, pH 6.5). Trace elements and high nitrate salt compositions were as described previously (71). For solid medium, 2% agar (wt/wt) was added. To grow the ΔKU80 pyrG1 strain, the medium was supplemented with 1.2 g/liter of uridine and uracil. When required, pyrithiamine (Sigma) or hygromycin B (Merck) was added to a final concentration of 0.2 or 300 μg/mL, respectively.

To induce heat stress in liquid cultures, 1 × 10^8^ conidia of the wild-type, *xylP*::*sdeA*, *xylP*::*hsfA*, *sdeA*::3×HA, and *xylP*::*hsfA sdeA*::3×HA strains were incubated in 50 mL of liquid MM (1% glucose) supplemented with xylose 1% for 24 h at 30°C to allow growth as described previously for other *xylP* conditional mutants (72). Subsequently, the mycelia were washed twice with prewarmed MM and incubated for 4 h at 30°C in MM xylose 1% (no glucose) or MM (1% glucose; no xylose) for induction or repression of the *xylP* promoter, respectively (36, 72). HS was induced by transferring the mycelia to fresh preheated MM (1% glucose) for an additional 15, 30, and 60 min of incubation at 48°C. The control was left at 30°C. Mycelia from each time point, from both pre- and post-HS exposure, were collected via vacuum filtration, flash-frozen in liquid nitrogen, and stored at −80°C until used for either RNA or protein extractions.

### Sequence analysis.

The following protein sequences were used to identify similar sequences at FungiDB (https://fungidb.org/fungidb/app/): Ole1 from S. cerevisiae (YGL055W), Ole1 from C. albicans (C1_08360C), Ole1 from S. pombe (SPCC1281.06c), SdeA from A. nidulans (An6731), SdeA from A. niger (An07g01960), SdeA from A. oryzae (AO090005000456), and human stearoyl-CoA desaturases SCD1 (NCBI ID 6319) and SCD5 (NCBI ID 79966). The selected sequences were downloaded and used in alignment and scoring protocols implemented in the Clustal Ω alignment tool (73) (http://www.ebi.ac.uk/Tools/msa/clustalo/). The graphical representations were performed using Jalview (2.10.1) software (74) (http://www.jalview.org). DeepTMHMM (75) (https://dtu.biolib.com/DeepTMHMM) was used for the prediction of transmembrane regions of SdeA, as previously reported (48).

### Strain construction.

All DNA cassettes were generated using a PCR-based strategy and *in vivo* recombination in yeast (71). For the *xylP*::*sdeA* cassette construction, two fragments from the 5′-untranslated region (5′-UTR) region of *sdeA* (AFUB_091500) and the *sdeA* gene were PCR-amplified from genomic DNA of the CEA17 strain according to Fig. S4A (https://doi.org/10.6084/m9.figshare.22355971). The primers used are listed in Table S2 (https://doi.org/10.6084/m9.figshare.22354573). The substitution cassette contained the promoter of the xylose reductase gene from P. chrysogenum (*xylP*) (37) and the *pyrG* gene as a prototrophy marker and was constructed as described previously (36). The cassette was transformed into protoplasts of the A. fumigatus ΔKU80 pyrG1 according to previously described procedures (71). Transformants were carefully tested by PCR with primers IM-319 and IM-440 (Fig. S4B [https://doi.org/10.6084/m9.figshare.22355971]) and Southern blot analysis (AlkPhos Direct Labeling and Detection System, Cytiva) using the 5′-flanking region as a probe and HindIII-digested genomic DNA (Fig. S4C [https://doi.org/10.6084/m9.figshare.22355971]).

To generate SdeA::GP fusion strain, a substitution cassette was constructed in which the *sdeA* genomic sequence without stop codon was cloned in-frame with the GFP gene in a C-terminal fusion (Fig. S4D [https://doi.org/10.6084/m9.figshare.22355971]). A C-terminal sequence of *sdeA* was amplified using the primers IM-320 and IM-321. The GFP::pyrG cassette (2.63 kb) was PCR-amplified from the plasmid pRS426+prx1::GFP (76) by using the primers IM-170 and IM-223. The amplification of the *sdeA* 3′-UTR region was performed with the primers IM-317 and IM-318. The *sdeA*::GFP cassette was generated by recombination in S. cerevisiae and transformed into the A. fumigatus wild-type strain. Transformants were carefully tested by PCR with primers IM-319 and IM-174 (Fig. S4E [https://doi.org/10.6084/m9.figshare.22355971]). Western blot using α-GFP antibody recognized the SdeA::GFP fusion protein at the expected size (Fig. S4F [https://doi.org/10.6084/m9.figshare.22355971]).

The same strategy was used to construct the *sdeA*::3×HA strain, in which the 3×HA tag was amplified from the pUC 3×HA *prtA* plasmid (kindly provided by Gustavo H. Goldman) by using the primers IM-193 and IM-329 (Fig. S4G [https://doi.org/10.6084/m9.figshare.22355971]). The *sdeA*::3×HA cassette was transformed into the wild-type and *xylP*::*hsfA* (36) strains to obtain single and double mutants. The pyrithiamine-resistant transformants were selected and tested by PCR with primers IM-319 and IM-329 (Fig. S4H [https://doi.org/10.6084/m9.figshare.22355971]) to confirm the *sdeA* locus replacement and Western blot analysis using an α-HA antibody (Fig. S4I [https://doi.org/10.6084/m9.figshare.22355971]).

To construct the double-tagged strain SdeA::GFP Cyp51A::monomeric red fluorescent protein (mRFP), the *cyp51A* (AFUB_063960) sequence without the stop codon was amplified using primers IM-700 and IM-655 from A1163 strain and cloned in-frame after the *gpdA* promoter amplified by the primers set IM-702 and IM-699 from the genomic DNA of A. nidulans GR5 strain. The *mRFP* gene was amplified from the AGB655 strain (kindly provided by Gustavo H. Goldman) (77). Finally, the 3×HA *prtA* cassette was again amplified from pUC 3×HA *prtA* using the primer IM-701 and IM-703 and cloned downstream the *mRFP* fragment. The cassette was amplified with the outermost primers IM-702 and IM-703 and transformed into the *sdeA*::GFP strain: The transformants were selected for pyrithiamine resistance.

### Biomass quantification in biofilms.

The quantification of biomass in the mature biofilms was performed as described by Gravelat et al. (78). In brief, 1 × 10^5^ conidia/mL were inoculated into 200 μL of MM (1% glucose) supplemented with xylose 1% in U-bottomed 96-well plates and allowed to grow for 20 h at 37°C. Next, the biofilm was washed three times with MM and incubated in MM (1% glucose) or MM supplemented with 1% xylose for four h at 37°C for *xylP* repression and induction, respectively. Following the incubation, the medium was removed, and the adhered mycelia were washed three times with sterile phosphate-buffered saline (PBS). A total of 150 μL of a 0.5% (wt/vol) crystal violet solution was added to each well for 5 min to stain the residual mycelia. Excess stain was gently removed by washing once with sterile water. The residual biofilm was destained with 200 μL of 95% ethanol per well for 16 h at room temperature. The biofilm density was measured by determining the absorbance of the destaining solution at 570 nm. The results were normalized by the fluorescence values of reduced resazurin (Sigma), which was used at a concentration of 10% in a separate and independent experiment to evaluate the growth of the strains and expressed as percentage of biomass. The experiments were performed in 12 technical replicates with at least three biological replicates. Statistical analysis was performed using two-way analysis of variance (ANOVA) with Sidak’s *post hoc* test (*P ≤ *0.05).

### XTT cell viability assay.

The XTT assay was performed as previously reported to measure the metabolic activity of mature A. fumigatus biofilms (79). A total of 1 × 10^5^ conidia/mL of the wild-type, *xylP*::*sdeA*, and *xylP*::*hsfA* strains were inoculated into 24-well plates containing 500 μL of MM (1% glucose) supplemented with 1% xylose and incubated at 37°C for 18 h. After growth, the culture medium was removed, and the wells were washed three times with MM. The biofilm formed was then incubated with MM for additional 4 h at 37°C. The control plates were kept at this condition, while cell wall stress was induced by adding 0.5 μg/mL of CASP for 15, 30, or 60 min. The culture medium was removed, and 300 μL of a 0.5 mg/mL XTT solution containing 50 μg/mL of vitamin K were added. The plates were left at 37°C for 1 h, and the absorbance of the resulting reduced XTT was read at 490 nm using the SpectraMax i3 microplate reader (Molecular Devices). The experiments were performed with three technical triplicates, and the data presented represent three biological replicates. Calculations were performed based on the basal values obtained from untreated biofilms for each strain. Statistical analysis was performed using two-way ANOVA with Sidak’s *post hoc* test (*P ≤ *0.05).

### Fluorescent microscopy.

Conidia of *sdeA*::GFP *cyp51A*::mRFP strain were inoculated in glass-bottomed dishes (MatTek Corporation) containing 2 mL of MM and incubated for 8 h at 37°C. Filter sets 38HE and 63HE (Carl Zeiss) were used to detect GFP and mRFP, respectively. Germlings were analyzed under a 100× magnification oil immersion objective (NA 1.4). The nuclei were visualized by staining with Hoechst (20 μg/mL). Images were captured with an AxioCam MRm camera coupled to a Zeiss Observer D.1 microscope and processed using ZEN software. For the SdeA::GFP relocation assays in the presence of antifungals, confocal laser scanning microscopy was performed using a Carl Zeiss LSM 800 confocal microscope with a Plan Apochromat 63×/1.40 Oil objective. The detection parameters for SdeA::GFP experiments were fixed as follows: laser, 488 nm; 35% 1.89 AU/84 μm detection wavelength, 488 to 574 nm; and detection gain, 850 V. The z-stack increments were 0.3 μm. The images were analyzed using ZEN Blue 2.3.

### Phenotypic assays.

The radial growth of the wild-type and *xylP*::*sdeA* strains at different temperatures was analyzed by spotting 1 × 10^4^ conidia of each strain in the center of 90-mm petri dishes containing solid MM (1% glucose) supplemented with various concentrations of xylose (0.25% to 5%). The plates were incubated for 72 h at 30, 37, or 48°C and analyzed. The same procedures were applied to investigate the radial growth of the strains in the presence of different FA. An aqueous solution containing 1% Igepal CA 630 was used for the FA solubilization in the MM, as previously described (30). The plates were incubated for 96 h at 37°C and photographed. Alternatively, 1 × 10^4^ conidia of each strain were inoculated in U-bottomed 96-well plates containing 200 μL of solid MM (1% glucose) supplemented with 2.5% xylose and different concentrations of aureobasidin A, cerulenin, trans-chalcone, myriocin, lovastatin, voriconazole, fluconazole, and AMB. The same procedures were followed for tests with the *xylP*::*hsfA* strain but using 0.06% xylose. Such xylose concentration allows the growth of the conditional lethal mutants and can evidence phenotypic differences among the strains grown in repressive conditions (1% glucose) in the presence of different stressing agents. All of the 96-well plates were incubated for 72 h at 37°C and analyzed.

### RNA extraction and RT-qPCR.

Flash-frozen mycelia from the HS cultures were disrupted by grinding in liquid nitrogen. The total RNA was extracted with TRIzol reagent (Thermo Scientific) according to the manufacturer’s protocol. RNA was purified, quantified, treated with Turbo DNase I (Thermo Scientific), and reverse-transcribed with a high-capacity cDNA reverse transcription kit (Thermo Scientific) as described previously (80). Quantitative reverse transcription PCR (RT-qPCR) was conducted with Power Sybr green PCR Master Mix (Thermo Scientific). The primers for the individual genes were designed using Primer Express 3.0 software (Life Technologies) and are listed in Table S3 (https://doi.org/10.6084/m9.figshare.22354573). RT-qPCR was performed in duplicate from three independent biological samples in a StepOne Plus real-time PCR system (Thermo Scientific). The fold change in mRNA abundance was calculated using 2^−ΔΔCt^ (81), and all the values were normalized to the expression of the A. fumigatus β-tubulin (*tubA*). Statistical analysis was performed using two-way ANOVA with Sidak’s *post hoc* test (*P ≤ *0.05).

### RNA sequencing.

To induce *hsfA* overexpression, 1 × 10^8^ conidia from wild-type and *xylP*::*hsfA* strains were incubated in 50 mL of liquid MM (1% glucose) supplemented with 1% xylose for 24 h at 30°C to allow growth. Subsequently, the mycelia were washed twice with MM 1% xylose and incubated for 4 h at 30°C in the same medium (xylose 1%; no glucose) for *hsfA* overexpression (36). Mycelium from each strain was collected via vacuum filtration, frozen in liquid nitrogen, and stored at −80°C. Total RNA was extracted using TRIzol reagent, treated with DNase I (Qiagen), and purified using the RNAeasy kit (Qiagen), according to the manufacturer’s instructions. Sample preparation, library construction, and data analysis were performed as described previously (36). Short reads were submitted to the NCBI’s Short Read Archive under Bioproject PRJNA690780. Gene ontology (GO) enrichment analysis was performed using the KOBAS tool (kobas.cbi.pku.edu.cn) (82).

### Protein extraction, immunoblotting, and co-IP procedures.

To assess the SdeA::3×HA expression, the mycelia obtained upon HS, according to the description above, were disrupted by grinding them in liquid nitrogen. Protein extraction and Western blotting were performed as before (80). The detection of SdeA::3×HA protein was achieved by using α-HA (H3663; Sigma) antibody according to the manufacturer’s instructions, as previously reported (83). To perform the co-IP assay, the *sdeA*::GFP strain and the GFP-Trap agarose resin (ChromoTek; GTA-20) were used as described elsewhere (84). The detection of SdeA::GFP protein was achieved by using α-GFP antibody (sc-9996; Santa Cruz Biotechnology). A. fumigatus Hsp90 was detected using a custom polyclonal α-Hsp90 antibody raised in rabbit (19). For α-HA and α-GFP detection, α-mouse IgG horseradish peroxidase (HRP) antibody (A4416; Sigma) was used, while α-rabbit IgG-HRP (Sigma; A0545) was the secondary antibody for α-Hsp90 detection. Chemoluminescent detection was performed by using an ECL Prime Western Blot detection kit (Cytiva). The images were generated by exposing the polyvinylidene difluoride (PVDF) membranes to the ChemiDoc XRS gel imaging system (Bio-Rad). The images were subjected to densitometric analysis in ImageJ software (85).

### Phospholipid and sphingolipid extraction.

For sphingolipid extraction, 300 mg of mycelia obtained upon HS according to the description above were suspended in Mandala buffer (86). Lipid extraction was carried out as described previously (87) with a few modifications. To disrupt the mycelia, the samples were vortexed and sonicated for 2 min in the presence of 0.2 mL of glass beads. The supernatant was collected, dried, and submitted to Bligh and Dyer extraction (88). One-third of each sample obtained after Bligh and Dyer extraction was reserved for inorganic phosphate (P_i_) determination, while the remaining was subjected to the alkaline hydrolysis of phospholipids (89). The same protocol was followed for phospholipid extraction but without the alkaline hydrolysis step.

### Sphingolipid mass spectrometry analysis.

Sphingolipid mass spectrometry analysis was carried out as previously reported (90). Briefly, a Thermo Accela high-performance liquid chromatography (HPLC) system (San Jose, CA) was used to separate the dried extracts dissolved in 150 μL of ammonium formate (1 mM) with 0.2% of formic acid in methanol. A Peeke Scientific Spectra C8 (Redwood City, CA) HPLC column (150 × 3 mm) was used, into which 10 μL of samples were injected. The HPLC was coupled to the HESI source of a Thermo TSQ Quantum Ultra triple quadrupole mass spectrometer (San Jose, CA). The sphingolipid profile was performed using positive ion mode, with the high voltage set to 3.5 kV, vaporizer temperature at 400°C, sheath gas pressure at 60, auxiliary gas pressure at 15, and a capillary temperature of 300°C. The collision cell was operated at 1.5 mTorr of argon. For the duration of the run, transitions for each lipid species were monitored at 100- or 50-ms dwell time. A total of 20 lipid standards for our profile from Avanti (Alabaster, AL) were used to develop calibration curves, and these curves were then used for lipid species to be monitored. Processing of the samples was done using Thermo Xcalibur 2.2 Quan browser software and exported to Excel for reporting results. Sphingolipid concentration determined by mass spectrometry was further normalized by the P_i_ abundance (91).

### Phospholipid mass spectrometry analysis.

Phospholipid mass spectrometry analysis was carried out as previously mentioned (92). The dried residue was reconstituted in 0.5 mL of the starting mobile phase solvent for LC-MS/MS analysis. Phospholipid classes were separated by reverse-phase LC using a Supelco 2.1 (inner diameter) × 150 mm Ascends Express C18 column (Sigma, St. Louis, MO) and a binary solvent system at a flow rate of 0.4 mL/min with a column oven set to 45°C. PC, PE, and PS were each quantified during each run by MRM analysis, and in addition, the structure was confirmed *via* a MS2 scan within the linear ion trap during the elution of each peak. For liquid chromatography-tandem mass spectrometry (LC-MS/MS) analyses, a Shimadzu Nexera LC-30 CE binary pump system coupled to a SIL-30 AC autoinjector and DGU20A5R degasser coupled to an AB Sciex 5500 quadrupole/linear ion trap (QTrap) (SCIEX Framingham, MA) operating in a triple quadrupole mode was used. Quarters 1 and 3 were set to pass molecularly distinctive precursor and product ions (or a scan across multiple *m*/*z* in quarter 1 or 3), using N_2_ to collisionally induce dissociations in quarter 2 (which was offset from quarter 1 by 30 to 120 eV); the ion source temperature was set to 500°C. The internal standards were l,2-diheptadecanoyl-*sn*-glycero-3-phosphocholine (17:0/17:0 PC), l,2-diheptadecanoyl-*sn*-glycero-3-phosphoethanolamine (17:0/17:0 PE) and l,2-diheptadecanoyl-*sn*-glycero-3-phospho-l-serine (17:0/17:0 PS). Phospholipids concentration determined by mass spectrometry was further normalized by the P_i_ abundance (91).

### Ergosterol extraction and quantification.

For sterol extraction, the same protocol used for sphingolipid extraction described above was followed, including the alkaline hydrolysis and the P_i_ estimation but without the addition of internal standards. Quantification was performed by thin layer chromatography (TLC). The ergosterol standard (Cayman Chemical) and the dried lipid samples were resuspended in 50 μL of chloroform/methanol (2:1 vol/vol), and 10 μL of each sample were spotted onto a silica gel plate (EMD Millipore) and run in a 10-foot × 10-foot glass TLC tank containing chloroform/methanol/water (65:25:4, vol/vol/vol) as a mobile phase prepared the day before. After 90 min of separation, the plates were dried at room temperature and developed with iodine crystals. Ergosterol was visualized by UV light (254 nm) for image acquisition. The images were subjected to densitometric analysis using the ImageJ software (85) and normalized to the P_i_ quantification.

### Data availability.

All the data herein described are included in the article.
